# Structure of VanS from vancomycin-resistant enterococci: A sensor kinase with weak ATP binding

**DOI:** 10.1016/j.jbc.2023.103001

**Published:** 2023-02-09

**Authors:** Kimberly C. Grasty, Claudia Guzik, Elizabeth J. D’Lauro, Shae B. Padrick, Joris Beld, Patrick J. Loll

**Affiliations:** 1 Department of Biochemistry and Molecular Biology, Drexel University College of Medicine, Philadelphia, Pennsylvania, USA; 2 Department of Microbiology and Immunology, Drexel University College of Medicine, Philadelphia, Pennsylvania, USA

**Keywords:** antibiotic resistance, vancomycin-resistant enterococci, two-component system, histidine kinase, ATP binding, CA, catalytic ATP-binding domain, DHP, dimerization and histidine-phosphorylation, PDB, Protein Data Bank, TCEP, Tris(2-carboxyethyl)phosphine, VRE, vancomycin-resistant enterococci

## Abstract

The VanRS two-component system regulates the resistance phenotype of vancomycin-resistant enterococci. VanS is a sensor histidine kinase that responds to the presence of vancomycin by autophosphorylating and subsequently transferring the phosphoryl group to the response regulator, VanR. The phosphotransfer activates VanR as a transcription factor, which initiates the expression of resistance genes. Structural information about VanS proteins has remained elusive, hindering the molecular-level understanding of their function. Here, we present X-ray crystal structures for the catalytic and ATP-binding (CA) domains of two VanS proteins, derived from vancomycin-resistant enterococci types A and C. Both proteins adopt the canonical Bergerat fold that has been observed for CA domains of other prokaryotic histidine kinases. We attempted to determine structures for the nucleotide-bound forms of both proteins; however, despite repeated efforts, these forms could not be crystallized, prompting us to measure the proteins' binding affinities for ATP. Unexpectedly, both CA domains displayed low affinities for the nucleotide, with *K*_*D*_ values in the low millimolar range. Since these *K*_*D*_ values are comparable to intracellular ATP concentrations, this weak substrate binding could reflect a way of regulating expression of the resistance phenotype.

For many years after its introduction, vancomycin occupied an important niche as a last-resort antibiotic, reserved for patients with beta-lactam allergies or infections refractory to other antibiotics. However, in the latter part of the 20th century, antibiotic-resistant pathogens such as methicillin-resistant *Staphylococcus aureus* began spreading rapidly, which elicited wider usage of vancomycin (1). Inevitably, this increased use led to increased levels of resistance. In particular, vancomycin resistance has now become commonplace in enterococci, transforming these organisms from largely benign commensals to vancomycin-resistant enterococci (VRE), serious pathogens that have been flagged by the World Health Organization as high-priority targets for therapeutic development (2, 3). VRE are now counted among the so-called ESKAPE pathogens, which are responsible for the majority of hospital-acquired infections and can be very challenging to treat (4).

Vancomycin interferes with production of the bacterial cell wall. It does so by binding the d-Ala-d-Ala sequence found at the C-terminus of lipid II's muramyl peptide, thereby sequestering a critical precursor in peptidoglycan biosynthesis (5, 6). Bacteria become resistant to vancomycin by acquiring a suite of genes that encode various remodeling enzymes, which degrade d-Ala-d-Ala and replace it with d-Ala-d-Ser or d-Ala-d-Lac, neither of which is efficiently recognized by the antibiotic (7). Expression of the remodeling-enzyme genes is controlled by VanS and VanR, which form a two-component system that senses the presence of the antibiotic and transduces this signal (8). Multiple VRE genotypes have been identified, corresponding to the acquisition of similar but distinct vancomycin-resistance genes; these genotypes are denoted as VRE types A through N (9).

Two-component systems are ubiquitous in bacteria, fungi, and plants (10, 11, 12) and consist of a sensor and a response regulator. The sensor (VanS in this case) is typically a transmembrane histidine kinase that autophosphorylates on a conserved histidine residue in response to an appropriate signal; the phosphoryl group is then transferred to the response regulator (here, VanR). VanR is a transcription factor, and phosphorylation enhances its affinity for the promoter controlling expression of the remodeling genes (13). Thus, in the presence of vancomycin, VanS will autophosphorylate and subsequently transfer the phosphoryl group to VanR, thereby activating transcription of the genes encoding the remodeling enzymes.

The VanS sensor kinase is organized into several structural domains (Fig. 1*A*), which include a periplasmic sensor domain flanked by two transmembrane helices, a membrane-proximal HAMP domain, a dimerization-and-histidine-phosphorylation (DHp) domain, and a catalytic ATP-binding (CA) domain (14, 15, 16). The latter plays a dominant role in the autophosphorylation mechanism, as it contributes all the residues responsible for proper positioning of the nucleotide during catalysis (17). Since CA domains are essential for histidine kinase activity (and therefore for two-component signaling), it is not surprising that these domains are being actively explored as potential targets for antimicrobial therapeutics (18).

**Figure 1:**
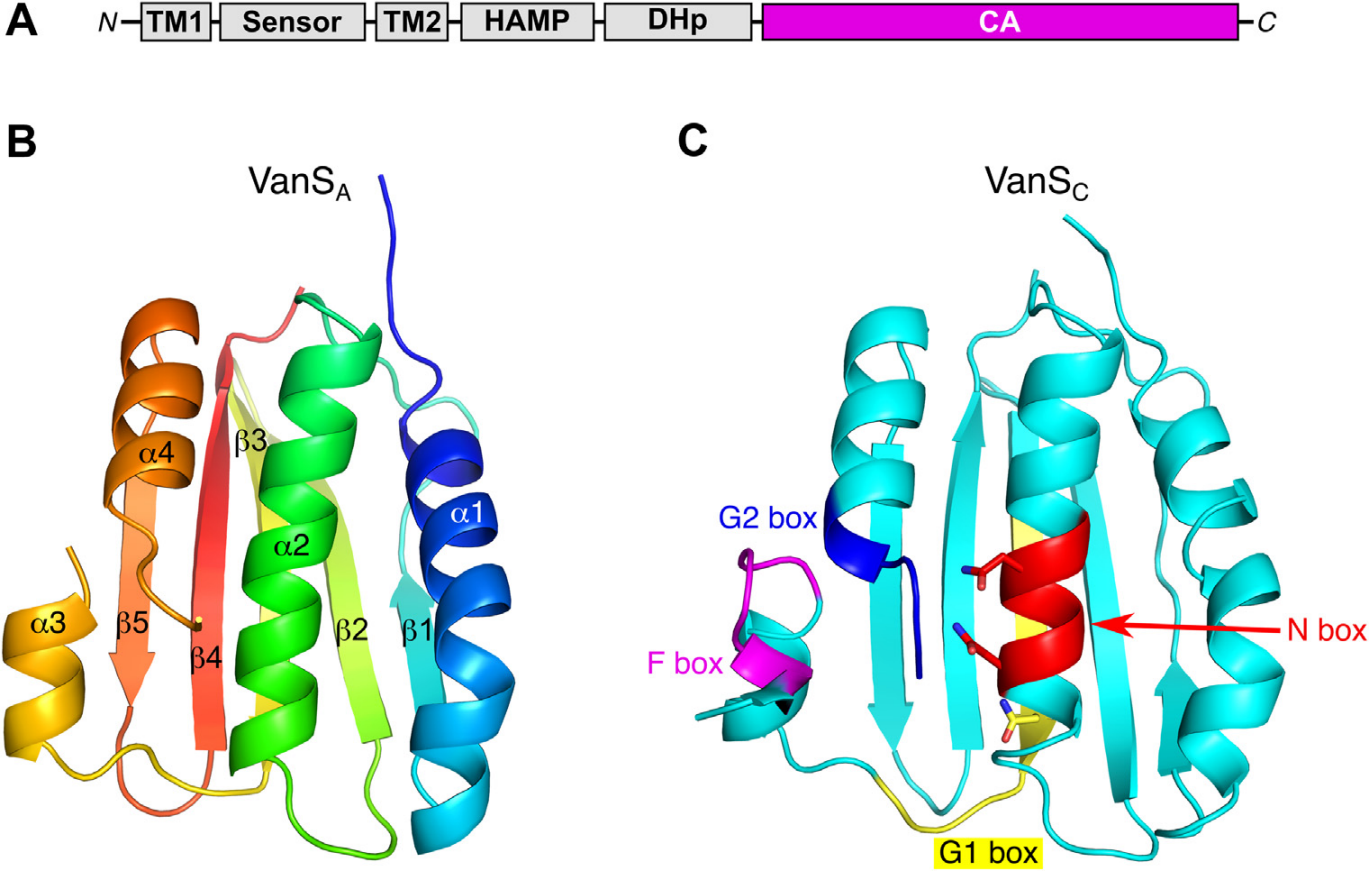
**Structures of the VanS**_**A**_**and VanS**_**C**_**CA domains.***A,* schematic illustration of the VanS domain structure. *B,* ribbon diagram showing the VanS_A_ CA-domain structure, colored using a “rainbow” scheme that ranges from *blue* at the N terminus to *red* at the C terminus. The numbering of the secondary-structure elements is also illustrated on the VanS_A_ structure (this numbering is the same for both VanS_A_ and VanS_C_). *C,* the VanS_C_ CA-domain structure, with key structural motifs highlighted (these motifs are present in both VanS_A_ and VanS_C_). These motifs include the N-box (*red*; the two conserved Asn residues are shown), the G1-box (*yellow*, including the noncanonical Asn residue found in both VanS_A_ and VanS_C_), the F-box (*magenta*), and the G2-box (*blue*). Stereo versions of *B* and *C* can be found in Fig. S12. CA, catalytic ATP-binding domain.

In the VanRS two-component system, structures are available for the inactive and activated forms of VanR (19), but comparable information is lacking for VanS. Thus, to gain further insight into the functioning of the VanRS system, we characterized the structures and functions of CA domains derived from two different VanS proteins. We report here the X-ray crystal structures of the VanS CA domains associated with type A and type C VRE. Unexpectedly, both CA domains were found to display unusually low affinities for their ATP substrate, raising intriguing questions about the potential contributions of nucleotide binding to the overall regulation of VanS activity.

## Results

### Structure determination of the VanS_A_ and VanS_C_ CA domains

As part of an effort to understand the molecular basis of vancomycin resistance in VRE, we purified and crystallized the CA domains of the VanS proteins associated with A-type and C-type resistance and determined their structures at resolutions of 2.2 and 1.45 Å, respectively (Table 1 and Figs. S1–S3). We will refer to the type A and type C proteins as VanS_A_ and VanS_C_.

**Table 1:** Data collection and refinement statistics

Data collection statistics	VanS_A_ 7 keV	VanS_A_ 13.5 keV	VanS_C_
PDB ID	8DWZ	8DVQ	8DX0
Diffraction source	Beamline 24-ID-C, Advanced Photon Source	Beamline 17-ID-1, NSLS-II	Beamline 24-ID-C, Advanced Photon Source
Wavelength (Å)	1.7712	0.92009	0.9791
Temperature (K)	100	100	100
Detector	Dectris Pilatus 6 MF	Eiger 9M	Dectris Pilatus 6 MF
Resolution range (Å)^a^	56.1–2.21 (2.29–2.21)	28.4–2.19 (2.27–2.19)	64.7–1.45 (1.50–1.45)
Spacegroup	*P*2_1_2_1_2	*P*2_1_2_1_2	*P*2_1_
Unit cell			
*a*, *b*, *c* (Å)	69.83, 94.40, 29.77	69.83, 94.40, 29.77	62.70, 40.23, 64.71
α, β, γ (°)	90.0, 90.0 90.0	90.0, 90.0 90.0	90.0, 91.64, 90.0
Total number of observations	176,601 (5296)	91,282 (8083)	345,447 (15,699)
Number of unique reflections	9612 (664)	10,676 (1031)	55,912 (4842)
Average multiplicity	18.4 (8.0)	8.55 (7.84)	6.2 (3.1)
Completeness (%)	92.8 (63.6)	99.7 (98.0)	97.1 (84.7)
Mean I/sigma(I)	23.1 (3.1)	13.0 (2.2)	22.8 (1.5)
Estimated Wilson *B*-factor (Å^2^)	40.4	43.4	21.9
R-merge^b^	0.101 (0.578)	0.090 (0.960)	0.036 (0.600)
R-meas^c^	0.104 (0.616)	0.096 (1.027)	0.039 (0.718)
R-pim^d^	0.023 (0.206)	0.033 (0.360)	0.016 (0.386)
CC_1/2_^e^	0.999 (0.932)	0.999 (0.849)	0.999 (0.682)
CC_1/2,anom_^e^	0.65 (0.10)	—	—
Resolution at which CC_1/2,anom_ drops below 0.3 (Å)	2.6	—	—
Refinement and model statistics			
Number of reflections used	17,544 (1807)	10,620 (1029)	51,113 (4534)
Reflections used for R-free	911 (98)	533 (45)	2630 (223)
Rwork	0.187 (0.268)^f^	0.192 (0.273)	0.185 (0.276)
Rfree	0.234 (0.328)^f^	0.239 (0.292)	0.200 (0.312)
Number of nonhydrogen atoms			
Protein	1006	1015	2245
Solvent	24	45	228
Ions	2	2	3
Average *B*-factor (Å)	55.6	63.0	28.1
RMSDs from ideality			
Bonds (Å)	0.007	0.01	0.01
Angles (°)	0.84	1.27	1.07
Residue distribution in Ramachandran plot			
Most favored region (%)	99.2	98.4	98.9
Allowed (%)	0.8	1.6	1.1
Outliers (%)	0	0	0
Clashscore	2.0	3.96	2.37

Data collection statistics were calculated using XDS (48) and the “Merging statistics” utility of the Phenix suite (49).

a Values in parentheses refer to the highest resolution shell.

b *R*_merge_ is calculated by the equation *R*_*merge*_*= Σ*_*hkl*_*Σ*_*i*_*|I*_*i*_*(hkl) - <I(hkl)>|/Σ*_*hkl*_*Σ*_*i*_*I*_*i*_*(hkl)*, where *I*_*i*_*(hkl)* is the *i*^th^ measurement.

c *R*_meas_ (or redundancy-independent *R*_merge_) is calculated by the equation *R*_*meas*_*= Σ*_*hkl*_*[N/(N - 1)]*^*½*^*Σ*_*i*_*|I*_*i*_*(hkl) - <I(hkl)>|/Σ*_*hkl*_*Σ*_*i*_*I*_*i*_*(hkl)*, where *I*_*i*_*(hkl)* is the *i*^th^ measurement and *N* is the redundancy of each unique reflection *hkl* (55).

d *R*_pim_ is calculated by the equation *R*_pim_*= Σ*_*hkl*_*[1/(N - 1)]*^*½*^*Σ*_*i*_*|I*_*i*_*(hkl) - <I(hkl)>|/Σ*_*hkl*_*Σ*_*i*_*I*_*i*_*(hkl)*, where *I*_*i*_*(hkl)* is the *i*^th^ measurement and *N* is the redundancy of each unique reflection *hkl* (56).

e *CC*_1/2_ is the correlation coefficient between two randomly chosen half data sets (57).

f For the VanS_A_ 7 keV data set, *F*(+) and *F*(−) were treated as separate observations during refinement.

The structure of the VanS_A_ CA domain was determined by the single-wavelength anomalous diffraction method, relying upon two ordered cadmium ions contributed by the crystallization buffer. The crystal asymmetric unit contains one copy of the CA domain, and the cadmium ions mediate two of the three lattice contacts that connect different VanS_A_ molecules within the crystal (Fig. S4). The structure was refined separately against two different data sets, one of which was measured at low energy (7 keV) and used for phasing, whereas the other was collected at an energy more typically used for protein crystallography (13.5 keV). The structures obtained from the two different data sets are essentially identical, with a few minor differences in side-chain conformers, and have an RMSD in Cα positions of 0.14 Å (0.59 Å for all nonhydrogen atoms).

The structure of the VanS_C_ CA domain was determined by molecular replacement, using an ensemble of homology models as probes. The crystal asymmetric unit contains two copies of the CA domain, related by an approximate twofold axis of symmetry. The two protomers in the asymmetric unit are highly similar, with superposition yielding an RMSD for Cα positions of 0.25 Å (0.74 Å for all atoms). Metal ions also figure in the crystal packing of the VanS_C_ protein, with 3 Mg^2+^ ions being situated in two different lattice interfaces (Fig. S5).

### Overall structure of the VanS CA domains

Both the VanS_A_ and VanS_C_ CA domains adopt the canonical Bergerat fold, which is associated with the GHKL superfamily of nucleotide-binding proteins, and in particular is characteristic of prokaryotic histidine kinases (14, 15). Like other CA domains, the VanS_A_ and VanS_C_ structures feature four alpha helices packed against one face of a five-stranded antiparallel beta sheet (Fig. 1). Also like other CA domains, the VanS structures contain a rare left-handed beta–alpha–beta crossover, corresponding to the β1–α2–β2 secondary structural elements. The presumptive nucleotide-binding site lies within a pocket bounded on either side by helices α2 and α4 and at the bottom by strands β3–5.

A series of conserved sequence motifs are associated with CA domains, namely the N, G1, F, and G2 boxes (20), and these motifs map to various positions clustered around the nucleotide-binding site. The N-box is found on helix α2 and derives its name from the second asparagine in the NxxxNA consensus sequence. This asparagine is highly conserved and typically coordinates the metal ion of the bound ATP–Mg^2+^ complex; it corresponds to Asn-281 in VanS_A_ and Asn-264 in VanS_C_.

The G1-box is found at the C-terminus of strand β3, which runs directly under helix α2. The canonical G1 consensus sequence is DxGxG. The aspartate residue of this sequence typically forms a salt bridge with the amine group of the nucleotide’s adenine base, whereas the first conserved glycine marks the point where the polypeptide chain makes a sharp ∼90° turn to run underneath the nucleotide-binding pocket. Interestingly, neither VanS_A_ nor VanS_C_ conform to this consensus sequence, having asparagine residues in place of the conserved aspartate, and lacking the second glycine altogether. Both deviations from the consensus are rare but not unprecedented. In particular, the Asp-to-Asn substitution is isosteric and, while it would eliminate the salt bridge, it should still allow for hydrogen bonding with the adenine base, and indeed the structure of the *Thermotoga maritima* kinase ThkA shows just such an asparagine–adenine hydrogen bond (21). The role of the second glycine in the G1-box is less clear, as it does not appear to contact the nucleotide in structures of CA domain–ATP complexes.

Immediately downstream of the G1-box lies an extended sequence that includes the short helix α3, followed by the other two conserved sequence motifs, the F-box and the G2-box. This portion of the CA domain is known as the ATP lid and is thought to close over the binding site once nucleotide is acquired. In the structures of both VanS_A_ and VanS_C_, a significant stretch of this extended region is disordered. It is common for part or all the ATP lids to be disordered in structures of nucleotide-free CA domains; in some cases, partial disorder remains even when nucleotides are bound (*e.g.*, Protein Data Bank [PDB] IDs 5C93 and 3SL2, corresponding to the kinases WalK and YycG). For both VanS_A_ and VanS_C_, the F-box is found just before the beginning of the disordered sequence, whereas the G2-box is found at the C-terminal end of the disordered region.

In addition to the motifs surrounding the nucleotide-binding site, CA domains also typically contain a conserved stretch of residues on the outward-facing surface of helix α4, immediately downstream of the G2-box. During autophosphorylation, these residues interact with the histidine kinase so as to properly position the CA domain, leading to α4 being known as the “gripper” helix (22). The conserved residues are arrayed along the outer face of the helix and are generally hydrophobic. In VanS_A_ and VanS_C_, these so-called “sticky-finger” residues are Ile-345, Ile-349, Gln-352 and Ile-328, Ile-332, Ala-335, respectively.

### Comparison of the VanS_A_ and VanS_C_ CA domains

**Figure 2:**
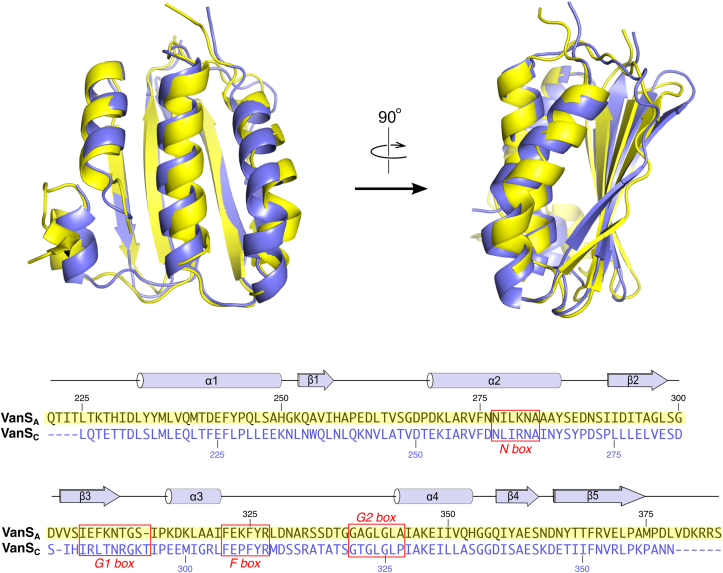
**Comparison of the VanS**_**A**_**and VanS**_**C**_**CA domains**. *Top*, superposition of VanS_A_ and VanS_C_, shown in two orthogonal orientations. VanS_A_ is colored *yellow*, whereas VanS_C_ is shown in *slate blue*. Below is shown the pairwise sequence alignment of the two CA domains. Secondary structural elements and conserved sequence motifs are indicated. CA, catalytic ATP-binding domain.

The sequences of the VanS_A_ and VanS_C_ CA domains are 37% identical, and both proteins adopt the same overall fold. To compare the two structures in more detail, we used TM-ALIGN to calculate a pairwise structural alignment (23). The VanS_A_ and VanS_C_ constructs contain 164 and 155 residues, respectively, and TM-ALIGN generated a superposition based upon 126 residues, with an RMSD value of 1.68 Å (Fig. 2). Pairwise superposition using the protein LSQKAB yields an RMSD value of 0.14 Å for alpha-carbon positions alone (24).

Overall, the two structures are very similar. The one exception can be found in the ATP lid; this is not surprising, given the flexible nature that is generally attributed to the ATP lids of CA domains. In particular, helix α3 occupies very different positions in the two structures, being positioned much closer to the nucleotide-binding site in VanS_A_ than in VanS_C_ (Fig. 2). Notably, α3 is involved in a crystal contact in the VanS_A_ crystals, packing against strand β5 in a neighboring molecule in the lattice (Fig. S4). Were α3 to occupy the same position in the VanS_A_ structure that it does in the VanS_C_ structure, it would clash with the symmetry-related molecule. Hence, it seems likely that the ATP lids of VanS_A_ and VanS_C_ can adopt a range of positions, and crystallization of these two proteins has selected for two different conformers.

### Nucleotide binding

Numerous efforts were made to cocrystallize the VanS_A_ and VanS_C_ CA domains with Mg^2+^ and nucleotide, using both ATP and the nonhydrolyzable analog AMP–PNP. However, no electron density for the nucleotide was ever seen with either protein. This failure to capture the nucleotide-bound forms prompted us to measure the CA domains’ binding affinities for ATP.

**Figure 3:**
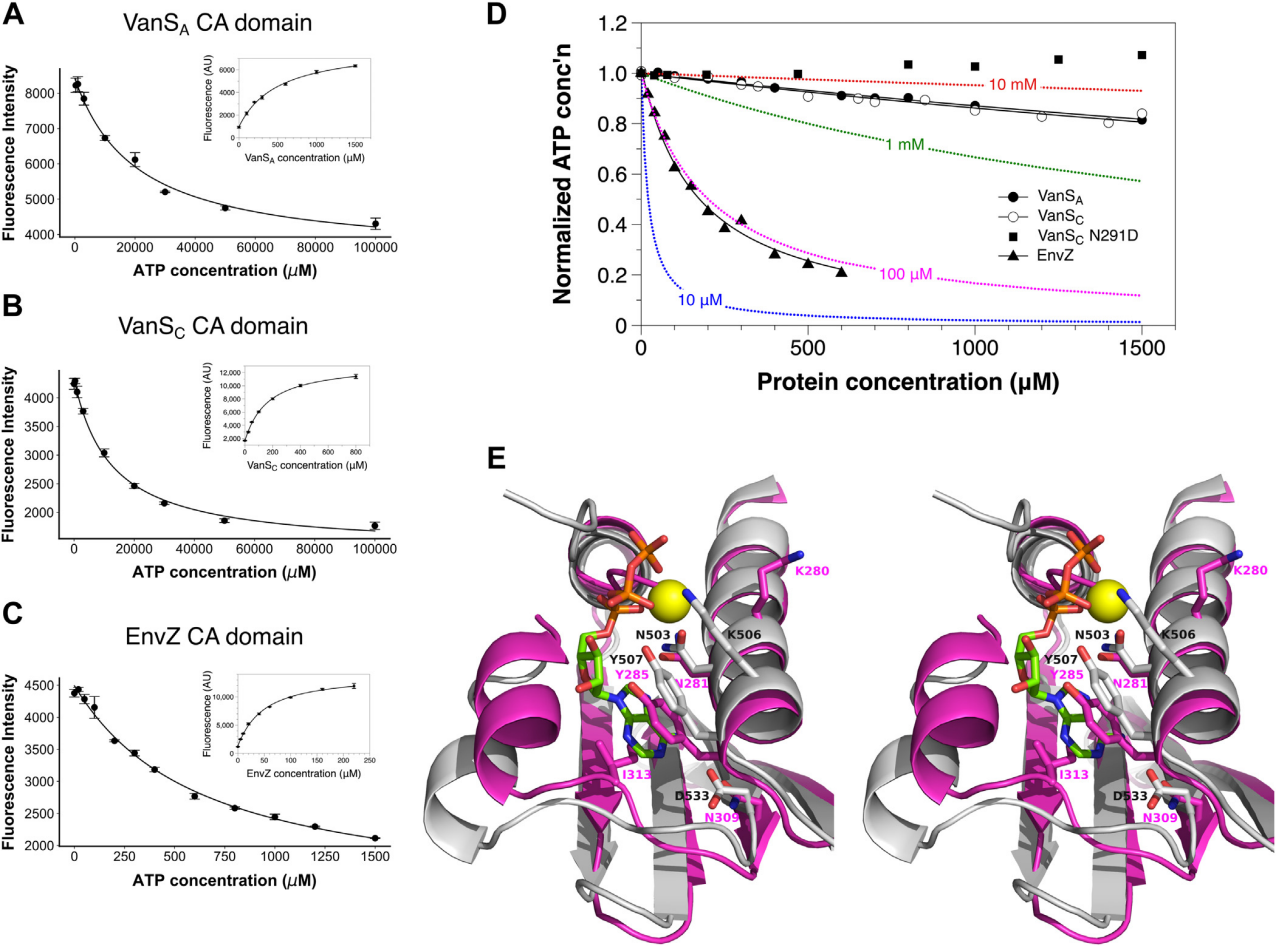
**Nucleotide binding by the VanS**_**A**_**and VanS**_**C**_**CA domains**. *A–C*, binding experiments using TNP–ATP. The *insets* show direct binding of TNP–ATP, whereas the main figures show competition assays in which TNP–ATP is displaced by ATP. *Lines* show fits of the appropriate binding expressions to the data. *A*, VanS_A_ CA domain; *B*, VanS_C_ CA domain; *C,* EnvZ CA domain. *D*, equilibrium dialysis binding experiments with ATP. *Solid black lines* represent fits of Equation 2 to the data. No attempt was made to fit a binding isotherm to the data for the N291D mutant of VanS_C_, since no evidence for binding was seen. *Dotted lines* in different colors show the theoretical binding curves for selected *K*_*D*_ values, as calculated from Equation 2. Dissociation constants inferred from the binding experiments in this figure are given in Table 2. *E*, divergent stereo view of the nucleotide-binding site of VanS_A_ (*magenta*), superimposed upon the ATP-bound structure of YycG from *Bacillus subtilis* (gray; Protein Data Bank ID: 3SL2). The magnesium ion in the YycG structure is shown as a *yellow sphere*. Selected amino-acid side chains in and around the nucleotide-binding site are shown. CA, catalytic ATP-binding domain.

We first used the fluorescent ATP analog TNP–ATP. This molecule’s emission profile is highly sensitive to the fluorophore’s environment, and thus it functions as a useful probe for protein binding (25, 26). As a positive control, we used the CA domain from *Escherichia coli* EnvZ, for which the TNP–ATP and ATP-binding affinities have previously been reported (27). Binding curves were measured for all three CA domains, yielding estimates for TNP–ATP affinity that lie between 40 and 500 μM (Table 2), which all fall within the range reported for TNP–ATP binding by other histidine kinases (27, 28, 29, 30). Next, ATP was used to displace TNP–ATP in competition experiments, allowing us to calculate *K*_*D*_ values for ATP (Fig. 3). The ATP-binding affinity obtained for the EnvZ CA domain was 290 μM, in reasonable agreement with previously published values, which range from 60 to 200 μM (27, 31). However, the estimated affinities for ATP were much weaker for the two VanS CA domains, falling in the low millimolar range (Table 2). Such *K*_*D*_ values are atypically high for histidine kinases (32).

**Table 2:** Nucleotide-binding affinities of different VanS constructs

Protein	TNP–ATP	ATP
	*K*_D_ (μM)	
EnvZ CA domain	43.2 ± 3.5	290.1 ± 38.5^a^ 83.3 ± 0.2^b^
VanS_A_ CA domain	499.6 ± 55.0	10,038 ± 1785^a^ 3948 ± 1.0^b^
VanS_A_ cytosolic domain	142.6 ± 6.4	7092 ± 788^a^
VanS_C_ CA domain	160.2 ± 10.3	6044 ± 655^a^ 3128 ± 0.4^b^
VanS_C_ N291D CA domain	870.7 ± 56.6	ND

a Value from TNP–ATP competition experiments.

b Value from equilibrium dialysis experiments.

To ensure that the low affinity for ATP was not an artifact associated with using the isolated CA domain, we repeated this experiment using the entire cytosolic domain of VanS_A_, which includes the HAMP and DHp domains. To prevent ATP consumption, we used the H164A mutant, which lacks the histidine that receives the autophosphorylation. While this cytosolic construct binds TNP–ATP roughly threefold more tightly than the isolated CA domain, its affinity for ATP is similar to that of the isolated CA domain (Fig. S6*A* and Table 2). Hence, it does not appear that weak ATP binding can be attributed to use of the isolated CA domains.

We next sought to confirm these results with an orthogonal ATP-binding assay. We chose to use equilibrium dialysis, exploiting the UV absorbance of the nucleotide to monitor free ATP concentration. Again, the *K*_*D*_ value we obtained for the EnvZ control protein fell within the range of previously published values (27, 31), providing confidence in the technique; and again, the binding affinities obtained for the two VanS CA domains were in the low millimolar range, in good agreement with the values obtained from the competition experiments (Fig. 3*D* and Table 2). Therefore, we can say with confidence that the CA domains of VanS_A_ and VanS_C_ proteins have unusually low affinities for ATP, which likely explains our inability to crystallize the protein–nucleotide complexes.

As discussed earlier, both the VanS_A_ and VanS_C_ CA domains contain asparagine in their G1-box sequences, rather than the more commonly occurring aspartic acid. Since this residue forms a salt bridge with the nucleotide’s primary amine in other CA domains, we questioned whether the Asp-to-Asn substitution was responsible for the abnormally low binding affinity of the VanS proteins. To test this idea, we made N309D (VanS_A_) and N291D (VanS_C_) mutants. However, this seemingly conservative change rendered VanS_A_ completely insoluble, both as the isolated CA domain and as the cytosolic-domain construct. The N291D VanS_C_ CA domain was also less stable than its wildtype form, but we were able to express and purify sufficient quantities to perform binding studies. The mutant’s affinity for TNP–ATP was decreased relative to wildtype (Fig. S6*B*); unfortunately, the ATP competition assay could not be performed, as protein precipitated in the presence of high concentrations of ATP. We were able to perform equilibrium dialysis experiments with the mutant, but these assays showed no evidence of ATP binding. Thus, while the interpretation of these experiments is complicated by the instability associated with the Asn-to-Asp mutations, they provide no evidence that restoring an aspartate to the VanS G1-box increases affinity for ATP. In addition, they suggest that this asparagine plays important roles in the VanS proteins’ folding and/or stability.

Finally, we tested whether VanS prefers GTP over ATP, as has been reported for another histidine kinase with the Asp-to-Asn substitution in its G1-box (33). Using the TNP–ATP competition assay, we compared the relative abilities of GTP and ATP to displace the fluorescent ATP analog and found that GTP bound less tightly than ATP to both VanS_A_ and VanS_C_ (Fig. S7), indicating that it is not the preferred substrate.

### Comparison with CA domains from other histidine kinases

**Figure 4:**
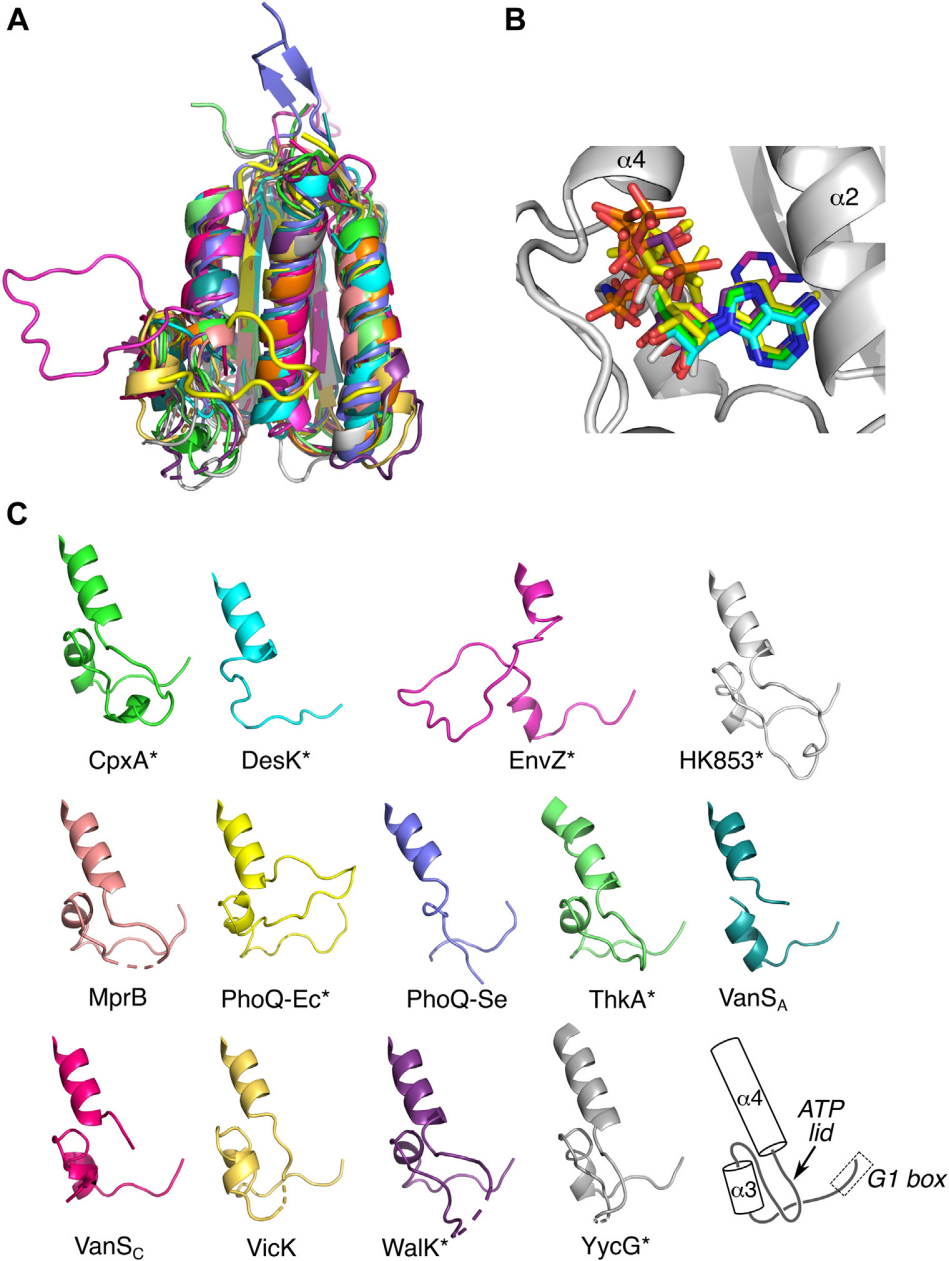
**Comparison of VanS**_**A**_**and VanS**_**C**_**with other prokaryotic CA domains.***A*, superposition of the VanS_A_ and VanS_C_ CA domains with 12 other CA domains, as performed using the program MULTIPROT (35). Details of the comparison structures can be found in Fig. S8. The different proteins adopt highly similar conformations, with the exception of the region between the G1-box and helix 4, which includes the ATP lid. This region lies at the *lower left* in this panel. *B*, comparison of the nucleotide conformations for the eight structures containing bound nucleotide. A structure cartoon for CpxA (*light gray*) is included to provide structural context. *C*, comparison of the region between the G1-box and helix α4. All structures were superimposed using MULTIPROT and are shown in the same orientation; the cartoon at the *lower right* illustrates the secondary structure elements involved. Color coding in this panel is the same as that used in *A*. Structures containing bound nucleotide are identified with an *asterisk*. The entire ATP lid is disordered in SrrB, which is therefore omitted from *C*. CA, catalytic ATP-binding domain.

To gain insight into possible factors controlling nucleotide affinity, and to place the VanS_A_ and VanS_C_ CA domains into the broader structural context of prokaryotic histidine kinases, the VanS structures were compared with those of 12 other CA domains. Structures in the comparison set included those of several well-studied CA-domain model proteins as well as the highest hits identified by BLAST searches using the two VanS CA-domain sequences. Both nucleotide-bound and nucleotide-free structures were included, with three containing no nucleotide, four containing a nucleotide but no associated metal ion, and five containing a nucleotide plus a metal ion (Fig. S8). MULTIPROT and SALIGN were used to calculate structural alignments for the 14 proteins (34, 35), with both programs giving essentially identical results. The alignment illustrates that the CA-domain fold is broadly conserved across all proteins considered, with the largest differences being found in the ATP lid (Fig. 4).

The length of the ATP-lid region differs among the proteins compared, ranging from 39 to 61 residues (for this calculation, the lid is defined as the segment connecting the end of the N-box with the beginning of the G2-box). The corresponding regions in VanS_A_ and VanS_C_ contain 58 and 57 residues, respectively. The largest conformational outlier in the lid region is EnvZ (PDB ID: 1BXD), for which the lid forms a loop extending away from the body of the protein. However, EnvZ is the only NMR structure included in the comparison, and its ATP lid was observed to be highly mobile, with a loop conformation that is likely poorly defined owing to a scarcity of medium- and long-range restraints (36). Portions of the ATP lid are also disordered for six of the remaining comparison structures, as well as for VanS_A_ and VanS_C_, all of which contain short regions between helices α3 and α4 that are undefined in the crystal structures. Apart from EnvZ, the other protein with a divergent ATP-lid conformation is *E. coli* PhoQ (PDB ID: 1ID0), in which the lid completely covers the nucleotide-binding site, extending from helix α4 all the way to α2. In the remainder of the structures, the ATP lid does not fully bury the nucleotide-binding site but rather runs alongside the site, leaving it partially accessible to solvent. In general (and perhaps somewhat surprisingly), there does not appear to be a correlation between the conformation of the ATP lid and the protein’s nucleotide-binding status; for example, *E. coli* PhoQ contains a Mg^2+^–AMPPNP complex, and its lid completely buries the bound nucleotide, but other structures that contain Mg^2+^–ATP do not assume the same lid conformation. Furthermore, the presence of a bound nucleotide is not sufficient to lock the ATP lid into a well-defined conformation, as several nucleotide-bound structures still display disorder in portions of their ATP lids (Fig. 4).

Helix α3 lies in the middle of the ATP lid region, and its position is roughly similar in all the compared structures, although in some proteins, it is partially unwound (*e.g.*, DesK and *Salmonella typhimurium* PhoQ). VanS_A_ is an exception, as its α3 helix lies closer to the nucleotide-binding site than in any of the other proteins; as described earlier, this likely reflects crystal packing effects.

We next sought to compare the ATP-binding sites of VanS_A_ and VanS_C_ with those of other CA domains, focusing on residues directly involved in binding the nucleotide. First, as noted earlier, most CA domains contain a conserved aspartic acid in the G1-box that forms a salt bridge with an amino group on the adenine base (14); in contrast, the two VanS proteins contain asparagine residues at this position, which adopt the same position and orientation typically associated with the aspartic acid. Therefore, these asparagines should in principle be capable of participating in a hydrogen bond with the base (Fig. 3*E*), and so it is not obvious that this substitution would radically alter nucleotide binding. Second, we considered the two hydrophobic residues (Tyr and Ile) that typically sandwich the base and observed that in the VanS CA domains, these residues are present and occupy positions that are similar to those seen in other CA domains. The same is true for a conserved asparagine in the N-box that helps coordinate the metal ion in the metal–nucleotide complex. Next, we noted that the phosphate groups of the nucleotide typically lie at the N-terminal end of helix α4, where their charge can be partially compensated by the helix dipole. The position of this helix is highly conserved in the various CA domains, including VanS_A_ and VanS_C_, although in the latter two structures, the polypeptide chain at the N-terminal end to the helix actually passes directly through the space that would be occupied by the nucleotide; presumably, this portion of the lid undergoes a rearrangement upon nucleotide binding. Finally, we observed a difference between the VanS proteins and the other CA domains at the C-terminal end of helix α2, immediately downstream of the N-box. In most CA domains, a lysine or an arginine is found here, which can form a salt-bridge interaction with the phosphate groups of the nucleotide. In VanS_A_ and VanS_C_, the equivalent position is occupied by an alanine and an asparagine, respectively, neither of which can make a comparable interaction. However, both VanS proteins contain a basic residue one helical turn upstream of this position, within the N-box (Lys-280 and Arg-263 in VanS_A_ and VanS_C_, respectively; Fig. 3*E*), which may compensate for the loss of the basic residue at the end of helix α2. Overall, then, while the nucleotide-binding sites of VanS_A_ and VanS_C_ differ slightly from those of other CA domains, there are no large structural changes that might explain a significant reduction in ATP affinity.

## Discussion

The VanS proteins play a critical role in the vancomycin-resistance mechanism, by sensing the presence of the antibiotic and, in response, regulating the expression of the resistance phenotype. To expand our understanding of VanS function, we have determined structures for the CA domains of two different VanS proteins, associated with types A and C VRE. Both proteins adopt the canonical Bergerat fold associated with histidine-kinase CA domains, even though they possess only modest sequence identity with many other CA domains. Our comparison of a panel of different CA domain–containing proteins reveals that all the proteins studied show strong structural similarity along their entire lengths, with the exception of their ATP-lid regions, which are conformationally diverse.

A surprising result to emerge from this work is the observation that the two VanS CA domains bind ATP with unusually low affinities, with both having *K*_*D*_ values in the low millimolar range. In contrast, other histidine-kinase CA domains that have been studied bind ATP roughly one to three orders of magnitude more tightly (17, 27, 28, 29, 30, 31, 37). Our affinity estimates are robust, as two orthogonal methods yield *K*_*D*_ estimates in agreement with each other (Table 2). It is worth noting that our affinity estimate for TNP–ATP binding to EnvZ is 20-fold weaker than previously reported (27); this may reflect the fact that the earlier work titrated TNP–ATP into a fixed concentration of protein, whereas we performed the opposite titration, in order to avoid complications from the inner-filter effect (25). Despite this difference, however, our estimates for EnvZ’s ATP-binding affinity agree well with published values.

The structures of the VanS_A_ and VanS_C_ CA domains do not offer immediate clues to explain their weak binding of ATP. Most of the residues associated with recognition of the nucleotide are either conserved in the two VanS proteins, or their functions can plausibly be performed by neighboring amino acids. One exception is a highly conserved aspartate normally found in the G1-box, which is replaced by asparagine in both VanS_A_ and VanS_C_. Attempts to produce the Asn-to-Asp mutant yielded unstable proteins in both cases, suggesting that this position may be important for protein folding in ways that are not currently clear. Whatever its role, this asparagine residue is highly conserved throughout different type A and type C isolates (Figs. S9 and S10). Indeed, the Asp-to-Asn variant is found in other VanS proteins as well, both from other VRE types and from nonenterococcal species (Fig. S11). Characterization of additional VanS CA domains will be required to ascertain whether this asparagine in the G1-box is invariably associated with low ATP affinity. If not, then another reasonable guess would be to ascribe the VanS proteins’ low affinity for ATP to the regions within the ATP lid that are not well conserved between VanS and other CA domains (Fig. S8).

It is conceivable that low affinities for ATP were observed because the CA domains behave differently in isolation than they do in the context of their full-length proteins. Arguing against this possibility is the observation that the complete cytoplasmic domain of VanS_A_ has a comparable ATP affinity to the isolated CA domain. However, the cytoplasmic-domain construct still lacks the membrane-binding and signal-recognition domains, which could affect nucleotide recognition. On the other hand, CA domains from many other histidine kinases recognize ATP with *K*_*D*_ values in the low micromolar range, demonstrating that using the isolated CA domains does not necessarily compromise nucleotide binding. Hence, it is plausible that the low affinities we have measured accurately reflect those of the full-length proteins, but this cannot be stated conclusively. Unfortunately, the biophysical assays necessary to directly address this question would require the preparation of many tens of milligrams of purified full-length VanS proteins, which is currently not feasible.

If the low ATP-binding affinities of the CA domains do, in fact, reflect the behavior of the full-length proteins, then the physiological implications are intriguing. Most kinases—including other histidine kinases—bind ATP with affinities in the 1 to 100 μM range, making the VanS proteins relative outliers (32, 38). The VanS proteins’ low affinities mean that their nucleotide-binding sites would essentially never approach saturation *in vivo*, since bacterial intracellular ATP concentrations typically fall in the 0.5 to 3 mM range (39, 40, 41, 42). This could represent a mechanism that helps minimize basal levels of autophosphorylation occurring in the absence of signal. Alternatively, matching ATP’s *K*_*D*_ with its cellular concentration could serve to make VanS activity sensitive to changes in ATP concentration, although the utility of regulating vancomycin resistance in this manner is not immediately apparent.

Finally, it is interesting to note that our two CA domains are derived from two different “flavors” of VRE, namely types A and C. Type A VRE exhibit inducible resistance to vancomycin, that is, cell wall precursors are not altered in the absence of the antibiotic; in contrast, many (but not all) type C VRE exhibit constitutive resistance, meaning that the resistance phenotype is observed in both the presence and absence of the antibiotic (9, 43). Such a difference would seem to imply a fundamental difference in the regulation of VanS activity between the two VRE types. As both types of VanS protein bind ATP with comparable affinities, it appears that any such difference will not be manifested in the actual autophosphorylation event but will more likely be found upstream of the ATP-binding or phosphorylation steps.

## Experimental procedures

### Construct preparation

The VanS_A_, VanS_C_, and EnvZ CA domains were expressed using the pETHSUL vector, which encodes the protein of interest fused with an N-terminally His_6_-tagged SUMO protein (44); the preparation of the cytosolic VanS_A_ construct was described previously (45). The sequence for the VanS_A_ CA domain (residues 221–384) was inserted into the pETHSUL expression vector using ligation-independent cloning, as described (44). The sequences for the VanS_C_ and EnvZ CA domains (residues 208–361 and 289–450, respectively) were inserted into pETHSUL *via* a DNA assembly reaction using the NEBuilder HiFi DNA assembly kit (New England Biolabs). After cleavage with SUMO hydrolase, the mature VanS_C_ and EnvZ CA domain proteins retain a single vector-derived glycine residue at their N termini, whereas the VanS_A_ CA domain does not. Primers used for subcloning are shown in Table S1.

### Expression and purification

The cytosolic VanS_A_ protein was expressed and purified as described (45). Expression constructs for the CA-domain proteins were transformed into BL-21 (DE3) competent cells (New England Biolabs). A single colony was picked and grown overnight at 37 °C in LB media containing 100 μg/ml ampicillin. The overnight culture was diluted 1:50 into 1 l of LB media containing 100 μg/ml ampicillin and placed in a shaking incubator at 30 °C. Once the absorbance reached a value of 0.6 to 0.8, the temperature was decreased to 20 °C and IPTG was added to a final concentration of 0.2 mM. The culture was shaken overnight at 225 rpm and harvested the following morning by centrifugation (3300*g*, 40 min). Cell pellets were stored at −80 °C before use.

All purification steps were performed at 4 °C. Cell pellets were thawed under cold running water and resuspended in buffer A (50 mM Tris [pH 7.8], 300 mM NaCl, 10 mM imidazole, and 0.1 mM Tris(2-carboxyethyl)phosphine [TCEP]) supplemented with 10 mM MgCl_2_ and 2 μg/ml each of DNase and RNase. The cells were lysed by three passes through an Emulsiflex C5 cell disrupter (Avestin). Cell lysates were clarified by sequential low- and high-speed centrifugation steps (15,300*g*, 30 min and 150,000*g,* 1 h, respectively). The clarified lysate was filtered through a 0.45-micron syringe filter and loaded onto a 5-ml IMAC HP column (GE Healthcare) equilibrated with buffer A. The column was then washed with 20 column volumes of buffer A and eluted with buffer B (50 mM Tris [pH 7.8], 300 mM NaCl, 250 mM imidazole, and 0.1 mM TCEP). To remove the fusion tag, the SUMO hydrolase dtUD1 (44) was added to the eluent (1 mg dtUD1/l of culture) and the mixture was dialyzed overnight in buffer A at 4 °C. The following day, the supernatant was passed through a 0.45-micron filter and loaded onto the 5-ml IMAC HP column and re-equilibrated with buffer A. The flow-through was collected, concentrated with an Amicon Ultra YM-10 concentrator (Millipore), and loaded onto an S100 26/60 size-exclusion column (GE Healthcare) equilibrated in 20 mM Tris (pH 7.8), 150 mM NaCl, 0.1 mM TCEP, and run at 1 ml/min. Both VanS CA domains eluted as single peaks at approximately 80 min. The purified proteins were concentrated and flash-cooled in liquid nitrogen and stored at −80 °C.

### Mass spectrometry

Proteins were analyzed on a Waters Acquity I-Class UPLC system coupled to a Synapt G2Si HDMS mass spectrometer in positive ion mode with a heated electrospray ionization source in a Z-spray configuration. LC separation was performed on a Waters Acquity UPLC Protein BEH C4 1.7 μm 2.1 × 50 mm column. For proteins, a 0.2 ml/min gradient of 80/20 to 30/70 A/B in 20 min followed by washing and reconditioning the column was used. Eluent A is 0.1% v/v formic acid in water and B is 0.1% v/v formic acid in acetonitrile. Conditions on the mass spectrometer were as follows: capillary voltage 0.5 kV, sampling cone 40 V, source offset 80 V, source 120 °C, desolvation 250 °C, cone gas 0 l/h, desolvation gas 1000 l/h, and nebulizer 6.5 bar. The analyzer was operated in resolution mode, and low-energy data were collected between 100 and 2000 Da at 0.2 s scan time. Electrospray ionization data were deconvoluted using MaxEnt1 in Masslynx 4.1 (Waters Corporation).

### Crystallization and structure determination

Crystals of the VanS_A_ CA domain were grown using protein at a concentration of 2.5 mg/ml in the size-exclusion buffer described above, supplemented with 2.5 mM AMP–PNP and 5 mM MgCl_2_. Protein and precipitant were mixed in a volume ratio of 1:2 and incubated under Al’s Oil at 18 °C (46); the precipitant solution was obtained from the Berkeley Screen (47) and contained 75 mM Bis–Tris propane, 25 mM citric acid, pH 8.2, 20 mM CdCl_2_, and 25% (w/v) PEG 400. Crystals were taken straight from the drop and flash-cooled in liquid nitrogen, with no added cryoprotectant.

Crystals of the VanS_C_ CA domain were grown using protein at a concentration of 2.5 mg/ml in size-exclusion buffer supplemented with 2.5 mM AMP–PNP and 5 mM MgCl_2_. Protein and precipitant were mixed in a volume ratio of 1:3 and incubated under Al’s Oil at 4 °C; the precipitant solution was 22% (w/v) PEG 6000 and 100 mM buffer. Crystals of comparable appearance and diffraction quality were obtained using MES buffer at pH 6.0, HEPES buffer at pH 7.0, and TRIS buffer at pH 8.0; the crystal used for the final data collection was grown at pH 7.0. Crystals were cryoprotected by step-wise transfer from the original mother liquor to a buffer containing 25% (v/v) 2,3-butanediol, 28% (w/v) PEG 6000, 200 mM CaCl_2_, 100 mM HEPES (pH 7.0), 2.5 mM AMP–PNP, and 20 mM MgCl_2_. Four steps were used, with approximately 3 min between transfers; after the final transfer, crystals were flash-cooled by plunging into liquid nitrogen.

Diffraction data were collected at beamlines 24-ID-C of the Advanced Photon Source and 17-ID-1 of the National Synchrotron Light Source II (NSLS-II); data were processed using XDS (48). The structure of the VanS_A_ CA domain was determined by single-wavelength anomalous diffraction phasing, using a wavelength of 1.771 Å (7 keV) to exploit the anomalous scattering of the cadmium ions present in the crystallization solution. Using the Autosol pipeline in Phenix, two cadmium positions were found, phases were calculated, and an initial model was built (49). The model was improved by alternating cycles of manual rebuilding in Coot (50) and refinement in Phenix. The initial model was refined separately against the 1.771 Å data set used for phasing and against another data set collected at a wavelength of 0.920 Å (13.5 keV). Both final models contain residues 228 to 375; no density was observed for residues 323 to 339 in the ATP lid.

The structure of the VanS_C_ CA domain was determined by molecular replacement. Homology models were built using SWISS-MODEL (51), and the top six models generated in this way were combined using the ENSEMBLER module of Phenix. The resulting probe molecule was positioned using PHASER to generate the initial model, which was subsequently improved by alternating cycles of manual rebuilding in Coot and refinement in Phenix. The final model contains residues 211 to 359; no density was observed for residues 312 to 323 in the ATP lid. Data collection and refinement statistics are shown in Table 1. PDB IDs for the structures presented herein are as follows: VanS_A_ CA domain (7 keV data), 8DWZ; VanS_A_ CA domain (13.5 keV data), 8DVQ; and VanS_C_ CA domain, 8DX0.

### Nucleotide binding

In order to measure the binding affinity for TNP–ATP, protein was dialyzed *versus* 50 mM Tris–Cl, pH 7.5, 50 mM KCl, and 5 mM MgCl_2_. Binding reactions were set up in 96-well plates, using a final volume of 100 μl. The TNP–ATP concentration was kept constant at 10 μM, while protein concentration was varied. Fluorescence was measured using a Tecan Spark plate reader fitted with a 510-nm dichroic mirror, using excitation/emission wavelengths of 440 and 545 nm, with bandwidths of 15 and 10 nm, respectively. Fluorescence data curves were fit to the following binding expression:

(1) $$F=F_{0}+\left(F_{\max}-F_{0}\right)\cdot \left[\frac{\left(K_{D}+T_{t}+P_{t}\right)-\sqrt{{\left(K_{D}+T_{t}+P_{t}\right)}^{2}-4P_{t}T_{t}}}{2P_{t}}\right]$$

where *T*_*t*_ and *P*_*t*_ are the total concentrations of TNP–ATP and protein, respectively.

For competition experiments, TNP–ATP concentration was kept constant at 10 μM, whereas the protein concentration was held constant at a value approximately equal to the *K*_*d*_ for TNP–ATP. Titration series were prepared using a stock solution of 150 mM Mg–ATP in 50 mM Tris–Cl, pH 7.5, 50 mM KCl, 5 mM MgCl_2_ (pH adjusted after nucleotide addition). Fluorescence measurements were conducted as described aforementioned. Competition data were fit to the exact competition expression derived by Wang (52).

Equilibrium-dialysis experiments were conducted using a SpectraPor instrument containing five dialysis cells. Each cell consisted of two halves, which were Teflon disks with shallow chambers. The two halves are used to construct a sandwich about a circular piece of 12 to 14 kDa molecular weight cutoff dialysis tubing, which separates the two chambers. To prepare for each experiment, protein was dialyzed overnight at 4° *versus* binding buffer (50 mM Tris [pH 7.5], 100 mM KCl, and 5 mM MgCl_2_). The five cells were inserted into the instrument, and for each cell, one chamber was filled with 300 μl of 140 μM ATP in binding buffer. The other chamber was filled with 300 μl of either binding buffer alone or various concentrations of protein in binding buffer. The five cells were then rotated at 4° for 20 to 24 h. Solutions in the ATP side of each cell were then removed, and ATP concentrations were determined by absorbance at 260 nM. The buffer absorbance in the buffer-only chamber was also measured in every experiment and was invariably found to be equal to that of the opposing, ATP-containing half of the cell, confirming that equilibrium had been achieved. This experiment required substantial quantities of purified protein because of the large volume of the sample chambers and the high concentrations required; therefore, we chose to measure many data points without replicates, rather than measuring a few data points in triplicate, in order to give the clearest picture of the shape of the binding curve. To provide a sense of the reproducibility of these measurements, data points were measured in random order, so that adjacent points on the curve were typically measured on separate days using different batches of protein. Dissociation constants were obtained by fitting the data to Equation 2 (the derivation of this expression is given in Supporting information section).

(2) $$L_{f}=\frac{\left(L_{0}-P-2K_{D}\right)+\sqrt{{\left(L_{0}-P-2K_{D}\right)}^{2}+8K_{D}\cdot L_{0}}}{4}$$

where *L*_0_ = initial concentration of ATP injected into the cell, *L*_f_ = concentration of free ATP at equilibrium, and *P* = the concentration of protein in the cell.

## Data availability

Coordinates and structure factors for the crystal structures reported herein can be found at the PDB (https://www.rcsb.org/), under the following entry IDs: 8DWZ (VanS_A_ CA domain, 7-keV data set); 8DVQ (VanS_A_ CA domain, 13.5-keV data set); and 8DX0 (VanS_C_ CA domain).

## Supporting information

This article contains supporting information (9, 23, 53, 54).

## Conflict of interest

The authors declare that they have no conflicts of interest with the contents of this article.
