# Supplementary material for: Structure of VanS from vancomycin-resistant enterococci: A sensor kinase with weak ATP binding
Source: J Biol Chem. 2023 Feb 9;299(3):103001. doi: 10.1016/j.jbc.2023.103001 (PMC10017428; doi:10.1016/j.jbc.2023.103001)
Supplement: Supplemental data [file mmc1.pdf]

Supporting Information

Structure of VanS from vancomycin-resistant Enterococci: A sensor kinase with weak ATP binding

Kimberly C. Grasty, Claudia Guzik, Elizabeth J. D'Lauro, Shae B. Padrick, Joris Beld, and Patrick J. Loll

List of Contents

Fig. S1. Purified proteins used in this work S2
Fig. S2. Representative $VanS_A$ electron density S3
Fig. S3. Representative $VanS_C$ electron density S4
Fig. S4. Crystal packing, $VanS_A$ CA domain S5
Fig. S5. Crystal packing, $VanS_C$ CA domain S6
Fig. S6. Nucleotide binding experiments, cytosolic $VanS_A$ & $VanS_C$ N291D S7
Fig. S7. Comparison of GTP and ATP binding S8
Fig. S8. Sequence alignment of selected CA domains. S9
Fig. S9. Sequence conservation within type-A VanS CA domains S10
Fig. S10. Sequence conservation within type-C VanS CA domains S11
Fig. S11. Conservation of the G1-box Asp-to-Asn variant S12
Fig. S12. Stereo images of the $VanS_A$ and $VanS_C$ CA-domain structures S13
Table S1. Primers used for preparation of expression constructs S14
Derivation. Binding expression for equilibrium-dialysis experiments S15

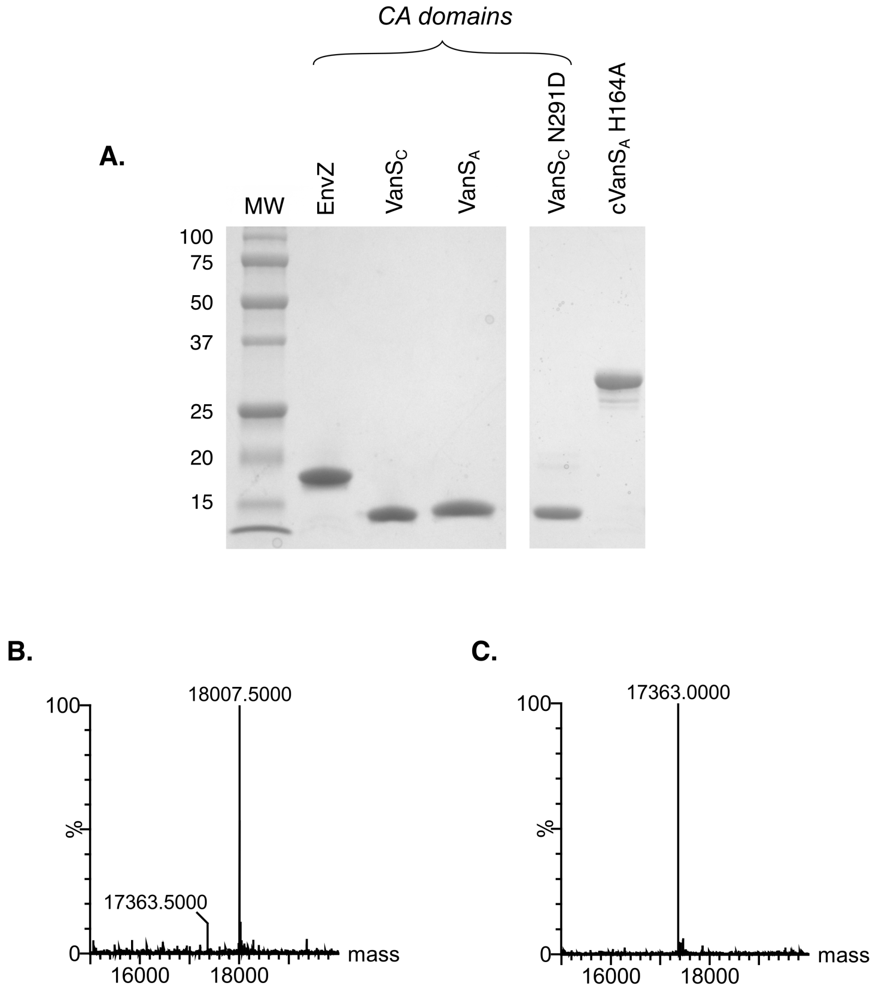

Figure S1: Purified CA-domain proteins. (A) Coomassie-stained SDS-PAGE gel showing purified proteins used in this work. The lane labeled "cVanS$_A$" shows the entire cytosolic domain of VanS$_A$. The lane labeled "MW" contains molecular-weight standards; values at left show the corresponding molecular weights, in kDa. Note that the VanS CA domains run anomalously on SDS-PAGE gels. (B & C) Deconvoluted mass spectra of the VanS CA-domain proteins, confirming the correct molecular masses. Panel (B) VanS$_A$ (calculated mass 18,008.3); Panel (C) VanS$_C$ (calculated mass 17,363.8). The small peak at 17363.5 Da in panel (B) represents a VanS$_C$ contaminant remaining from an earlier run.

A.

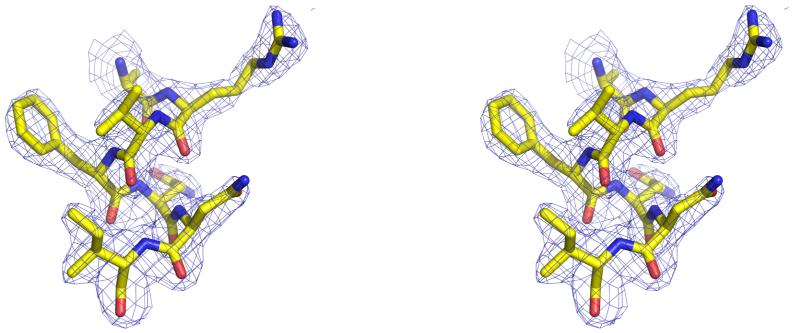

B.

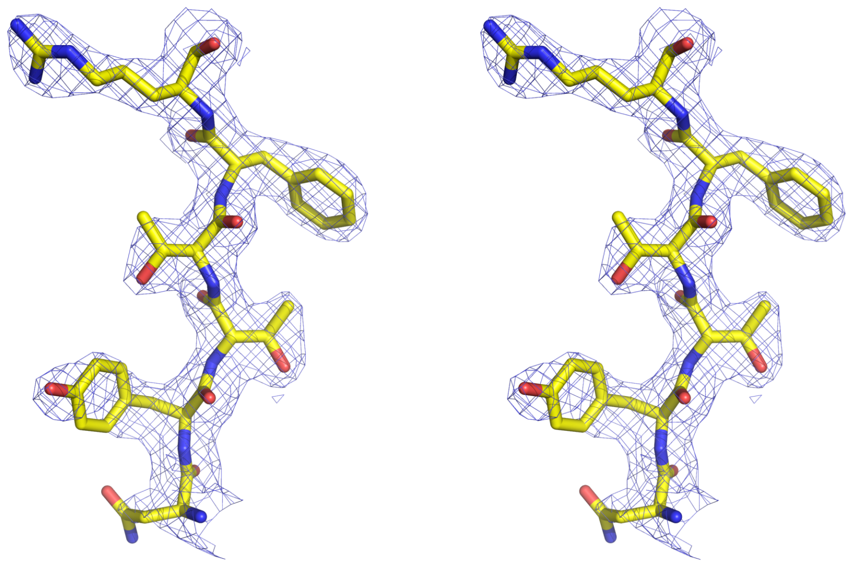

Figure S2: Representative VanS$_A$ electron density. Divergent stereo images of 2Fo-Fc electron density, contoured at 1.2σ. (A) Helix α2. (B) Strand β5.

A.

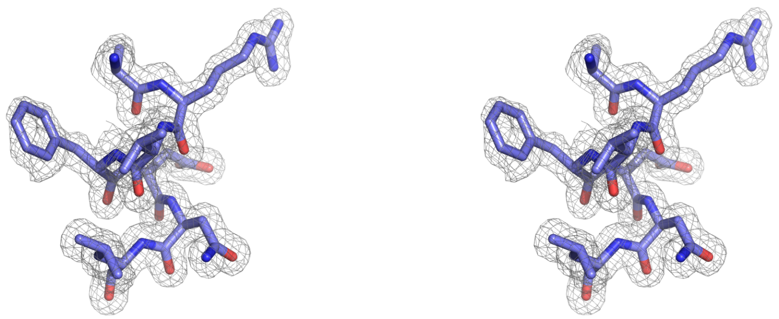

B.

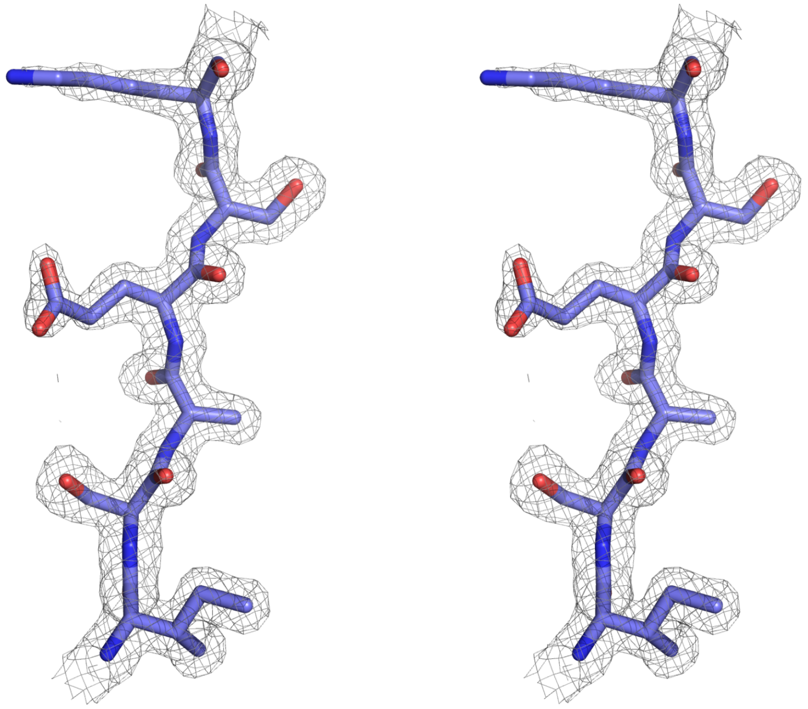

Figure S3: Representative VanS$_C$ electron density. Divergent stereo images of 2Fo-Fc electron density, contoured at 1.2σ. (A) Helix α2. (B) Strand β4.

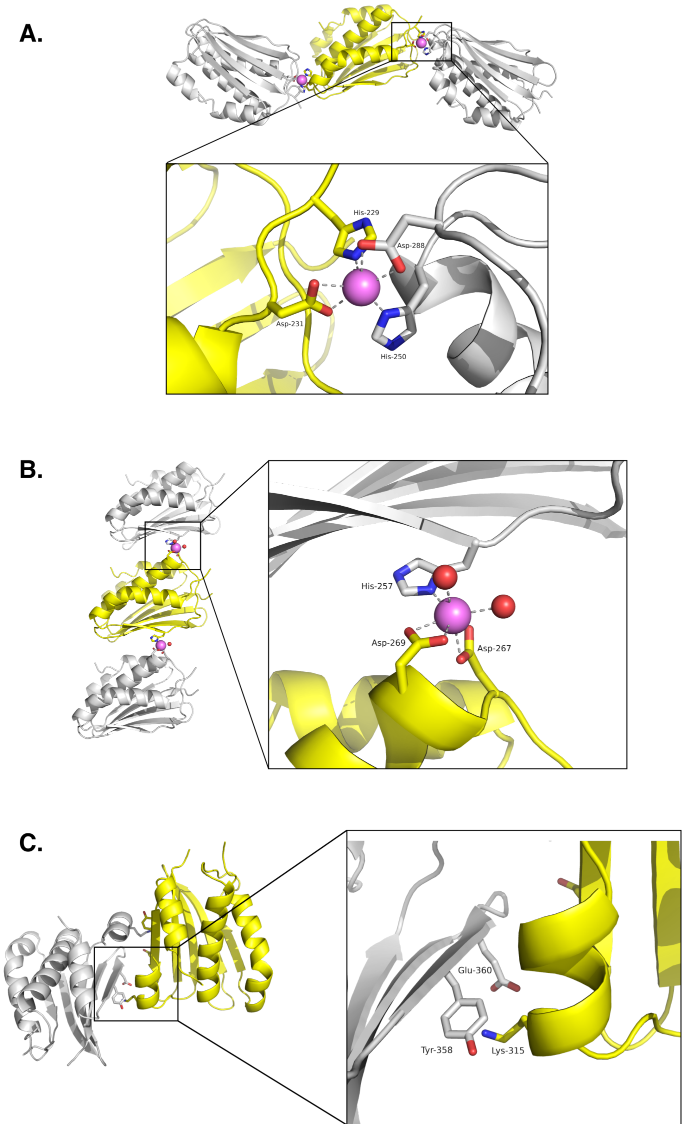

Figure S4: Lattice contacts in crystals of the VanS$_A$ CA domain. (A) One of two crystal contacts mediated by solvent-derived cadmium ions. A single VanS$_A$ protein is highlighted in yellow, with two symmetry-related molecules shown in gray; the cadmium ion is shown as a pink sphere. A similar color scheme is used in the other panels of this figure. (B) The second cadmium-mediated crystal contact. Water molecules are shown as small red spheres. (C) Third crystal contact, involving helix α3. Lys-315 interacts with either Tyr-358 or Glu-360 on a neighboring molecule (the interaction with Tyr-358 is seen in PDB ID 8DWZ, shown in this panel, while a salt bridge with Glu-360 is seen in 8DVQ). Helix α3 sits closer to the main body of the protein in the VanS$_A$ CA domain than it does in other CA-domain structures, likely due to this crystal contact. All three of the lattice contacts shown in this figure are reciprocal, meaning interactions contributed by one molecule are mirrored by corresponding interactions from the symmetry mate; hence, the three types of lattice contacts give rise to six actual contact points/monomer.

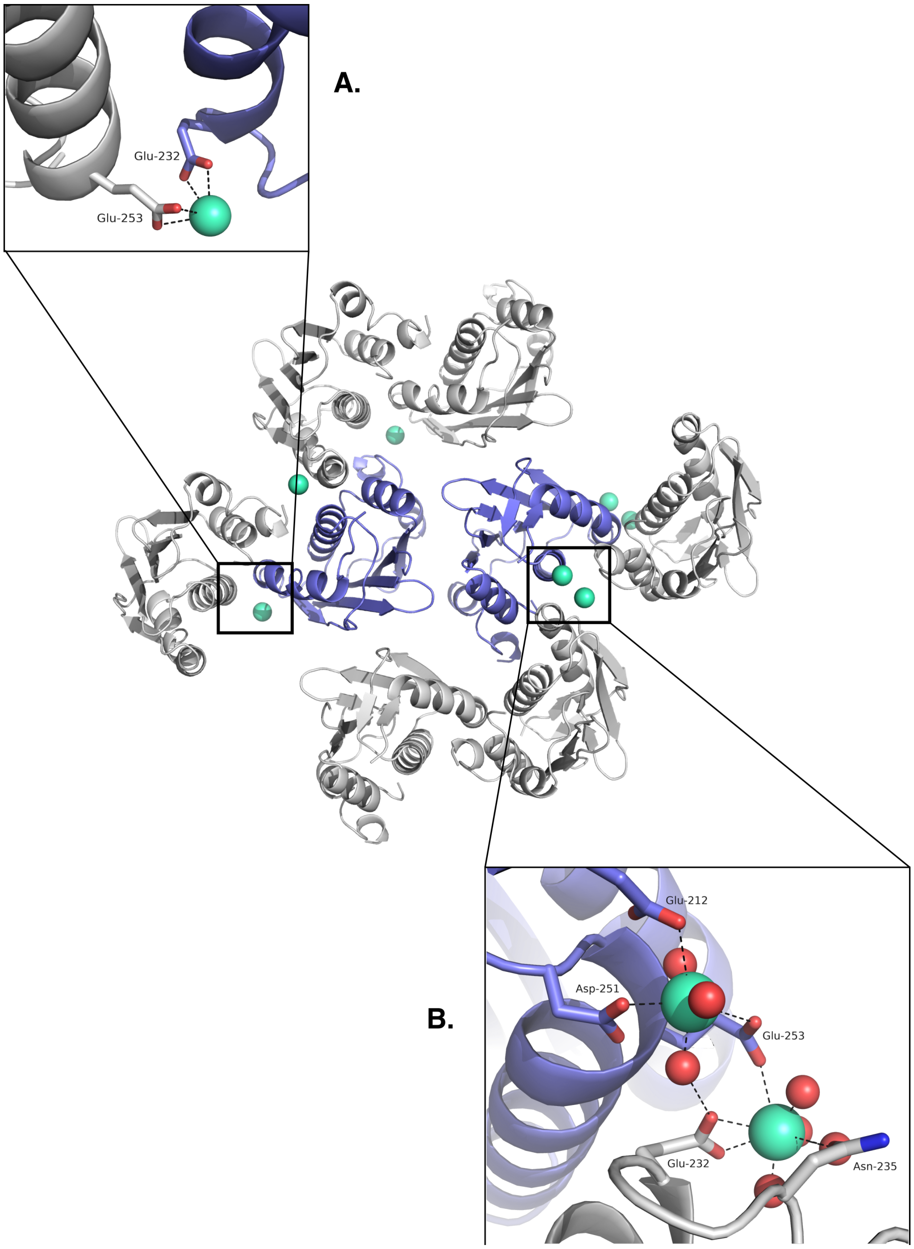

Figure S5: Lattice contacts in crystals of the VanS$_C$ CA domain. Two distinct magnesium-mediated contacts are seen. The asymmetric unit contains two VanS$_C$ chains (shown in blue); selected symmetry-related molecules are shown in gray. Some of the same residues in different chains participate in different contacts. (A) A simple contact involved shared coordination of a single magnesium ion. (B) A more complex contact involving two magnesium ions coordinated between two neighboring chains. Magnesium ions are shown as green spheres, and water molecules are shown as smaller red spheres.

A.

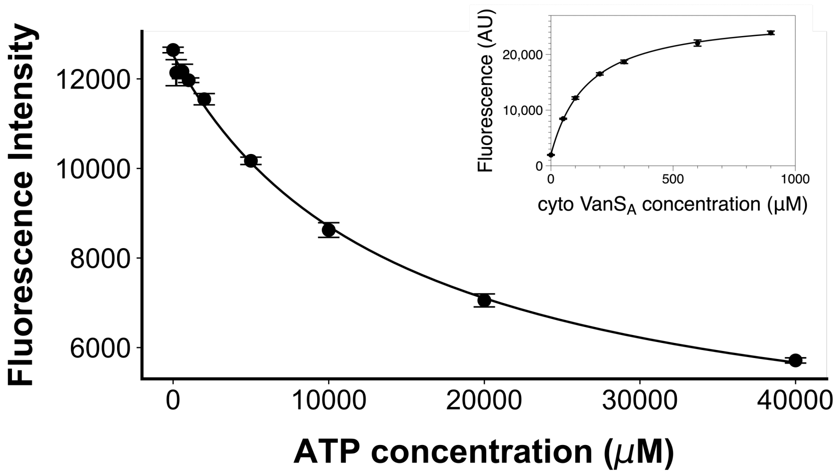

B.

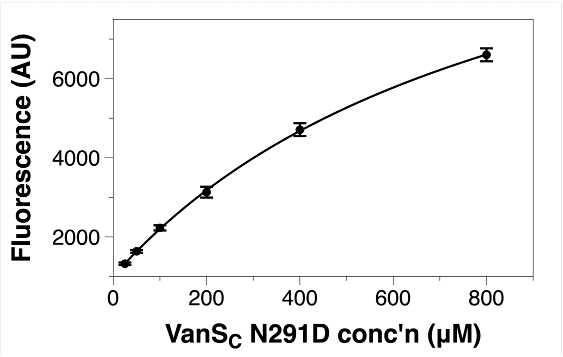

Figure S6: Nucleotide binding experimental controls. (A) Binding experiments using TNP-ATP and the cytosolic domain of $VanS_A$. The inset shows direct binding of TNP-ATP, while the main figure shows a competition assay in which TNP-ATP is displaced by ATP. (B) TNP-ATP binding by the N291D mutant of the $VanS_C$ CA domain. Lines show fits of the appropriate binding expressions to the data. Dissociation constants inferred from the binding experiments shown in this figure are given in Table 2.

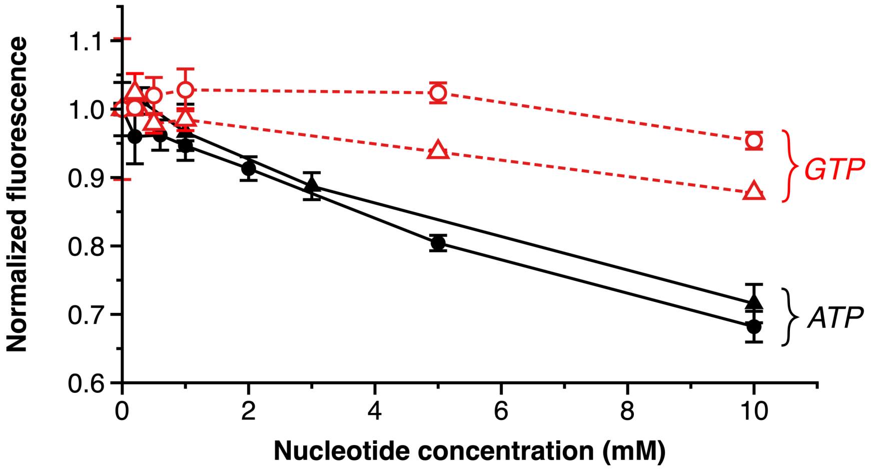

Figure S7. Comparison of GTP vs. ATP binding. The TNP-ATP competition assay was used to compare GTP binding with ATP binding. Circles correspond to the cytosolic $VanS_A$ construct, and triangles correspond to the $VanS_C$ CA domain. Filled black symbols represent the competition data obtained with ATP (these data are also shown in Figure 3, panel B, and in Figure S6, panel A); open red symbols show the competition data for GTP. For both constructs, GTP is significantly less effective than ATP at displacing TNP-ATP.

```
                                                                                                    N-box                                      G1-box
VanSA        QTITLTKTHIDLYYMLVQMTDEFYPQ---LSAHGKQAVIHAP---EDLTVSGDPDKLARVFNNILKNAAAYSED---NSIIDITAGL---SGDVVSIEFKNTGS-IPKDK
VanSC        ----LQTETTDLSLMLEQLTFEFLPL---LEEKNLNWQLNLQ---KNVLATVDTEKIARVFDNLIRNAINYSYP---DSPLLLELVE---SDS-IHIRLTNRGKTIPEEM
3A0T ThkA    ----MEFTEFNLNELIREVYVLFEEK---IRKMNIDFCFETD--NEDLRVEADRTRIKQVLINLVQNAIEATGE---NGKIKITSED-MYTK--VRVSVWNSGPPIPEEL
3EHG DesK    -GIRLKDELINIKQILEAADIMFIYE---EEKWPENISL-LN---ENI--------LSMCLKEAVTNVVKHSQA----KTCRVDIQQ--LWKE-VVITVSDDGT------
1BXD EnvZ    TGQEMPMEMADLNAVLGEVIAAESG-----YEREIETALYPG--S--IEVKMHPLSIKRAVANMVVNAARYG-----NGWIKVSSGT-EPNR--AWFQVEDDGPGIAPEQ
3DGE HK385   KSLQINREKVDLCDLVESAVNAIKEF---ASSHNVNVLFESNVPCPVEAY-IDPTRIRQVLLNLLNNGVKYSKKDAPDKYVKVILDE-KDGG--VLIIVEDNGIGIPDHA
1ID0 PhoQ Ec ------RELHPVAPLLDNLTSALNKV---YQRKGVNISLDIS--PE-ISFVGEQNDFVEVMGNVLDNACKYCL-----EFVEISARQ-TDEH--LYIVVEDDGPGIPLSK
3CGZ PhoQ St -SVLLSRELHPVAPLLDNLISALNKV---YQRKGVNISMDIS--PE-ISFVGEQNDFVEVMGNVLDNACKYCL-----EFVEISARQ-TDDH--LHIFVEDDGPGIPHSK
3SL2 YycG    -------MWIQIVRFMSLIIDRFEMT------KEQHVEFIRNLPDRDLYVEIDQDKITQVLDNIISNALKYSPE---GGHVTFSIDV-NEEEELLYISVKDEGIGIPKKD
4I5S VicK    QTSHLDVELTNFTAFMNYILDRFDQI-QSQQSTNKVYEIIRDYPDKSVWIEIDTDKMTQVIDNILNNAIKYSPD---GGKVTITMQT-TDTQ--LILSISDQGLGIPKKD
5C93 WalK    GTTRVDMELVNINEMFNYVLDRFDMILKKDDNPAKYYTIKREFTKRDLWVEIDTDKFTQVLDNIMNNAIKYSPD---GGVVTCRLLE-THNQ--VIISISDQGLGIPRAD
6PAJ SrrB    EGLSVNKEVQPIAALLDKMKIKYRQQ---ADDLGLNMTFNY---CKKRVWSYDMDRMDQVLTNLIDNASRYTK---PGDEIAITCDE-NESE--DILYIKDTGTGIAPEH
4BIV CpxA    KNALVS-ETIKANQLWSEVLDNAAFE---AEQMGKSLTVNFP-PGPWPLY-GNPNALESALENIVRNALRYSH-----TKIEVGFAV-DKDG--ITITVDDDGPGVSPED
6BLK MprB    -GAMVVHEPVDMTEVIDRSLERVRR-----RRSDIEFEVTVT--P--WQVIGDSSGLGRAVLNVLDNAAKWSPP---GGRVGVRLYQIDPGH--AELVITDQGPGIPPQE

                 F-box          G2-box
VanSA        LAAIFEKFYRLDNARSSDTGGAGLGLAIAKEIIV-QHGGQIYAESNDNY-TTFRVELPAMPDLVDKRRS---
VanSC        IGRLFEPFYRMDSSRATATSGTGLGLPIAKEILL-ASGGDISAESKDET-IIFNVRLPKPANN---------
3A0T ThkA    KEKIFSPFFTTKT------QGTGLGLSICRKIIEdEHGGKIWTENRENG-VVFIFEIPK--------TPEKR
3EHG DesK    -------FKGEENSFSK----GHGLLGMRERLE-FANGSLHIDTE-NG-TKLTMAIPNNSK----------
1BXD EnvZ    RKHLFQPFVRGDSART--ISGTGLGLAIVQRIVD-NHNGMLELGTSERGGLSIRAWLPV-PVTRAQGTTKEG
3DGE HK385   KDRIFEQFYRVDSSLTYEVPGTGLGLAITKEIVE-LHGGRIWVESEVGKGSRFFVWIP--KDRAGEDNRQDN
1ID0 PhoQ Ec REVIFDRGQRVDTLR----PGQGVGLAVAREITE-QYEGKIVAGESMLGGARMEVIFGR-----QHSAPKDE
3CGZ PhoQ St RSLVFDRGQRADTLR----PGQGVGLAVAREITE-QYAGQIIASDSLLGGARMEVVFGR-----QHPTQKEE
3SL2 YycG    VEKVFDRFYRVDKARTRKLGGTGLGLAIAKEMVQ-AHGGDIWADSIEGKGTTITFTLPYK--EEQEDDWDEA
4I5S VicK    LPLIFDRFYRVDKARSRAQGGTGLGLAIAKEIVK-QHKGFIWANSEEGEGSTFTIVLPYENDNDAIDEWEED
5C93 WalK    LGHVFDRFFRVDKARSRAQGGTGLGLAISKEVVQ-MLGGRIWVDSVEGKGSTFYISLPYEPYEEE-DLWDDD
6PAJ SrrB    LQQVFDRFYKVDAARTRGKQGTGLGLFICKMIIE-EHGGSIDVKSELGKGTTFIIKLP--KPE---------
4BIV CpxA    REQIFRPFYRTDEARDRESGGTGLGLAIVETAIQ-QHRGWVKAEDSPLGGLRLVIWLP--LYKRS-------
6BLK MprB    RHLVFERFFRSASARS--MPGSGLGLAIVKQVVL-KHGGALRVDYADPAGTAIHIVLPGRPM----------
```

Protein	Organism	PDB ID	Ligand	RMSD vs. VanS$_A$ (Å)	Sequence identity with VanS$_A$ (%)	RMSD vs. VanS$_C$ (Å)	Sequence identity with VanS$_C$ (%)
VanS$_A$	*Enterococcus faecium*	8DWZ	none	---	100	1.68	37.4
VanS$_C$	*Enterococcus gallinarum*	8DX0	none	1.68	37.4	---	100
ThkA	*Thermotoga maritima*	3A0T	Mg-ADP	2.09	24.8	1.80	22.7
DesK	*Bacillus subtilis*	3EHG	Mg-ATP	2.71	14.6	2.74	14.9
EnvZ	*Escherichia coli*	1BXD	AMPPNP	2.71	21.3	2.49	22.1
HK385	*Thermotoga maritima*	3DGE	ADP	1.89	25.6	1.94	29.4
PhoQ-Ec	*Escherichia coli*	1ID0	Mg-AMPPNP	2.05	22.1	2.29	20.4
PhoQ-Se	*Salmonella enterica, subsp. enterica, serovar Typhimurium*	3CGZ	none	2.30	21.4	2.40	20.1
YycG	*Bacillus subtilis*	3SL2	Mg-ATP	1.94	30.5	1.83	26.6
VicK	*Streptococcus mutans*	4I5S	none	2.06	33.1	1.83	29.0
WalK	*Lactiplantibacillus plantarum*	5C93	AMPPCP	2.25	29.6	1.82	26.4
SrrB	*Staphylococcus aureus*	6PAJ	none	1.75	28.3	1.61	25.2
CpxA	*Escherichia coli*	4BIV	ATP	2.37	25.0	2.00	21.4
MprB	*Mycolicibacterium hassiacum*	6BLK	Mg-ATP	1.97	22.9	1.86	20.4

Figure S8. Top, alignment of the VanS$_A$ and VanS$_C$ CA-domain sequences with those of the twelve homologs shown in Figure 4. Highly conserved residues are shown in red, and moderately conserved residues in blue. The locations of key sequence motifs are indicated. The Asp-to-Asn substitution that occurs in the G1 box of the VanS proteins is highlighted in yellow. Bottom, details of the various homolog structures. Alignment and sequence identities were calculated with COBALT (53); RMSD values were calculated for pairwise alignments using the program TM-align (23). Citations refer to reference numbers in the main text of this article.

```
                      221                                                                   N box
VanS (this work)      QTITLTKTHIDLYYMLVQMTDEFYPQLSAHGKQAVIHAPEDLTVSGDPDKLARVFNNILKNAAAYSEDNS
WP_025478673.1        QTITLTKTHIDLYYMLVQMTDEFYPQLSAHGKQAVIHAPEDLTVSGDPDKLARVFNNILKNAAAYSEDNS
ELB79878.1            QTITLTKTHIDLYYMLVQMTDEFYPQLSAHGKQAVIHAPEDLTVSGDPDKLARVFNNILKNAAAYSEDNS
WP_013438845.1        QTITLTKTHIDLYYMLVQMTDEFYPQLSAHGKQAVIHAPEDLTVSGDPDKLARVFNNILKNAAAYSEDNS
KUE81969.1            QTITLTKTHIDLYYMLVQMTDEFYPQLSAHGKQAVIHAPEDLTVSGDPDKLARVFNNILKNAAAYSEDNS
EOD92576.1            QTITLTKTHIDLYYMLVQMTDEFYPQLSAHGKQAVIHAPEDLTVSGDPDKLARVFNNILKNAAAYSEDNS
EOF35106.1            QTITLTKTHIDLYYMLVQMTDEFYPQLSAHGKQAVIHAPEDLTVSGDPDKLARVFNNILKNAAAYSEDNS
EOL66494.1            QTITLTKTHIDLYYMLVQMTDEFYPQLSAHGKQAVIHAPEDLTVSGDPDKLARVFNNILKNAAAYSEDNS
WP_169032517.1        QTITLTKTHIDLYYMLVQMTDEFYPQLSAHGKQAVIHAPEDLTVSGDPDKLARVFNNILKNAAAYSEDNS
WP_152139220.1        QTITLTKTHIDLYYMLVQMTDEFYPQLSAHGKQAVIHASEDLTVSGDPDKLARVFNNILKNAAAYSEDNS
WP_025191924.1        QTITLTKTHIDLYYMLVQMTDEFYPQLSAHGKQAVIHAPEDLTVSGDPDKLARVFNNILKNAAAYSEDNS
AOO95692.1            QTITLTKTHIDLYYMLVQMTDEFYPQLSAHGKQAVIHAPEDLTVSGDPDKLARVFNNILKNAAAYSEDNS
WP_100908275.1        QTITLTKTHIDLYYMLVQMTDEFYPQLSAHGKQAVIHAPEDLTVSGDPDKLARVFNNILKNAAAYSEDNS
AKE81061.1            QTITLTKTHIDLYYMLVQMTDEFYPQLSAHGKQAVIHAPEDLTVSGDPDKLARVFNNILKNAAAYSEDNS
ADA62246.1            QTITLTKTHIDLYYMLVQMTDEFYPQLSAHGKQAVIHAPEDLTVSGDPDKLARVFNNILKNAAAYSEDNS
WP_001810399.1        QTITLTKTHIDLYYMLVQMTDEFYPQLSAHGKQAVIHAPEDLTVSGDPDKLARVFNNILKNAAAYSEDNS
WP_002305818.1        QTITLTKTHIDLYYMLVQMTDEFYPQLSAHGKQAVIHAPEDLTVSGDPDKLARVFNNILKNAAAYSEDNS
                      ************************************** *******************************

                      291           G1 box          F box           G2 box
VanS (this work)      IIDITAGLSGDVVSIEFKNTGSIPKDKLAAIFEKFYRLDNARSSDTGGAGLGLAIAKEIIVQHGGQIYAE
WP_025478673.1        IIDITAGLSGDVVSIEFKNTGSIPKDKLAAIFEKFYRLDNARSSDTGGAGLGLAIAKEIIVQHGGQIYAE
ELB79878.1            IIDITAGLSGDVVSIEFKNTGSIPKDKLAAIFEKFYRLDNARSSDTGGAGLGLAIAKEIIVQHGGQIYAE
WP_013438845.1        IIDITAGLSGDVVSIEFKNTGSIPKDKLAAIFEKFYRLDNARSSDTGGAGLGLAIAKEIIVQHGGQIYAE
KUE81969.1            IIDITAGLSGDVVSIEFKNTGSIPKDKLAAIFEKFYRLDNARSSDTGGAGLGLAIAKEIIVQHGGQIYAE
EOD92576.1            IIDITAGLSGDVVSIEFKNTGSIPKDKLAAIFEKFYRLDNARSSDTGGAGLGLAIAKEIIVQHGGQIYAE
EOF35106.1            IIDITAGLSGDVVSIEFKNTGSIPKDKLAAIFEKFYRLDNARSSDTGGAGLGLAIAKEIIVQHGGQIYAE
EOL66494.1            IIDITAGLSGDVVSIEFKNTGSIPKDKLAAIFEKFYRLDNARSSDTGGAGLGLAIAKEIIVQHGGQIYAE
WP_169032517.1        IIDITAGLSGDVVSIEFKNTGSIPKDKLAAIFEKFYRLDNARSSDTGGAGLGLAIAKEIIVQHGGQIYAA
WP_152139220.1        IIDITAGLSGDVVSIEFKNTGSIPKDKLAAIFEKFYRLDNARSSDTGGAGLGLAIAKEIIVQHGGQIYAE
WP_025191924.1        IIDITAGLSGDVVSIEFKNTGSIPKDKLAAIFEKFYRLDNARSSDTGGAGLGLAIAKEIIVQHGGQIYAE
AOO95692.1            IIDITAGLSGDVVSIEFKNTGSIPKDKLAAIFEKFYRLDNARSSDTGGAGLGLAIAKEIIVQHGGQIYAE
WP_100908275.1        IIDITAGLSGDVVSIEFKNTGSIPKDKLAAIFEKFYRLDNARSSDTGGAGLGLAIAKEIIVQHGGQIYAE
AKE81061.1            IIDITAGLSGDVVSIEFKNTGSIPKDKLAAIFEKFYRLDNARSSDTGGAGLGLAIAKEIIVQHGGQIYAE
ADA62246.1            IIDITAGLSGDVVSIEFKNTGSIPKDKLAAIFEKFYRLDNARSSDTGGAGLGLAIAKEIIVQHGGQIYAE
WP_001810399.1        IIDITAGLSGDVVSIEFKNTGSIPKDKLAAIFEKFYRLDNARSSDTGGAGLGLAIAKEIIVQHGGQIYAE
WP_002305818.1        IIDITAGLSGDVVSIEFKNTGSIPKDKLAAIFEKFYRLDNARSSDTGGAGLGLAIAKEIIVQHGGQIYAE
                      *********************************************************************

                      361
VanS (this work)      SNDNYTTFRVELPAMPDLVDKRRS
WP_025478673.1        SNDNYTTFRVELPGFCCKVLNKE-
ELB79878.1            SNDNYTTFRVELPGFCCKVLNKE-
WP_013438845.1        SNDNYTTFRVELPAMPDLVDKRRS
KUE81969.1            SNDNYTTFRVELPAMPDLVDKRRS
EOD92576.1            SNDNYMTFRVELPAMPDLVDKRRS
EOF35106.1            SNDNYTTFRVELPAMPDLVDKRRS
EOL66494.1            SNDNYTTFRVELPAMPDLVDKRRS
WP_169032517.1        SNDNYTTFRVELPAMPDLVDKRRS
WP_152139220.1        SNDNYTTFRVELPAMPDLVDKRRS
WP_025191924.1        SNDNYMTFRVELPAMPDLVDKRRS
AOO95692.1            SNDNYTTFRVELPAMPDLVDKRRS
WP_100908275.1        SNDNYTTFRVELPAMPDLVDKRRS
AKE81061.1            SNDNYTTFRVELPAMPDLVDKRRS
ADA62246.1            SNDNYTTFRVELPAMPDLVDKRRS
WP_001810399.1        SNDNYTTFRVELPAMPDLVDKRRS
WP_002305818.1        SNDNYTTFRVELPAMPDLVDKRRS
                      ***** *******.:   * ::.
```

Figure S9. Sequence conservation within type-A CA-domain sequences. Shown is the Clustal Omega alignment (54) of sixteen $VanS_A$ sequences (9) taken from the NCBI Identical Protein Groups database (https://www.ncbi.nlm.nih.gov/ipg). Also shown is the sequence corresponding to the structure reported in this paper; the numbering shown is for the latter structure. Asterisks indicate 100% sequence conservation at a given position; two dots indicate conservation including residues with highly similar properties; and one dot indicates conservation with residues with weakly similar properties.

```
                         208                                                    N box
VanS_C (this work)       LQTETTDLSLMLEQLTFEFLPLLEEKNLNWQLNLQKNVLATVDTEKIARVFDNLIRNAINYSYPDSPLLL
WP_081116391.1           LQTETTDLSLMLEQLTFEFLPLLEEKNLHWQLQLQKKVFATVDTEKMERVFDNLIRNAINYSYPNSPLRL
EOH76242.1               LQTETTDLSLMLEQLTFEFLPLLEEKNLHWQLQLQKKVFATVDTEKMERVFDNLIRNAINYSYPNSPLRL
WP_016608740.1           LQTETTDLSLMLEQLTFEFLPLLEEKNLHWQLQLQKKVFATVDTEKMERVFDNLIRNAINYSYPNSPLRL
EEV40576.2               LQTETTDLSLMLEQLTFEFLPLLEEKNLHWQLQLQKKVFATVDTEKMERVFDNLIRNAINYSYPNSPLRL
WP_005228833.1           LQTETTDLSLMLEQLTFEFLPLLEEKNLHWQLQLQKKVFATVDTEKMERVFDNLIRNAINYSYPNSPLRL
WP_034869172.1           LQTETTDLSLMLEQLTFEFLPLLEEKNLHWQLQLQKKVFATVDTEKMERVFDNLIRNAINYSYPNSPLRL
WP_142968727.1           LQTETTDLSLMLEQLTFEFLPLLEEKNLHWQLQLQKKVFATVDTEKMERVFDNLIRNAINYSYPNSPLRL
GEB29600.1               LQTETTDLSLMLEQLTFEFLPLLEEKNLHWQLQLQKKVFATVDTEKMERVFDNLIRNAINYSYPNSPLRL
WP_156236642.1           LQTETTDLSLMLEQLTFEFLPLLEEKNLHWQLQLQKKVFATVDTEKMERVFDNLIRNAINYSYPNSPLRL
WP_142964727.1           LQTETTDLSLMLEQLTFEFLPLLEEKNLHWQLQLQKKVFATVDTEKMERVFDNLIRNAINYSYPNSPLRL
WP_142973537.1           LQTETTDLSLMLEQLTFEFLPLLEEKNLHWQLQLQKKVFATVDTEKMERVFDNLIRNAINYSYPNSPLRL
WP_142437529.1           LQTETTDLSLMLEQLTFEFLPLLEEKNLHWQLQLQKKVYATVDTEKMERVFDNLIRNAINYSYPNSPLHL
WP_142962782.1           LQTETTDLSLMLEQLTFEFLPLLEEKNLHWQLQLQKKVFATVDTEKMERVFDNLIRNAINYSYTDSPLHL
WP_081118442.1           LQTETTDLSLMLEQLTFEFLPLLEEKNLHWQLQLQKKVFATVDTEKMERVFDNLIRNAINYSYTDSPLHL
WP_140965761.1           LQTETTDLSLMLEQLTFEFLPLLEEKNLHWQLQLQKKVFATVDTEKMERVFDNLIRNAINYSYTDSPLHL
WP_096742870.1           LQTETTDLSLMLEQLTFEFLPLLEEKNLHWQLQLQKKVFATVDTEKMERVFDNLIHNAINYSYADSPLHL
OOG26042.1               LQTETTDLSLMLEQLTFEFLPLLEEKNLHWQLQLQKKVFATVDTEKMERVFDNLIRNAINYSYADYPLHL
WP_161642824.1           LQTETTDLSLMLEQLTFEFLPLLEEKKLHWQLQIQKKVFATVDTEKMERVFDNLIRNAINYSYPDSPMSL
WP_086269313.1           LQTETTDLSLMLEQLTFEFLPLLEEKNLNWQLNLQKNVLATVDTEKIARVFDNLIRNAINYSYPDSPLLL
WP_155853362.1           LQTETTDLSLMLEQLTFEFLPLLEEKNLNWQLNLQKNVLATVDTEKIARVFDNLIRNAINYSYPDSPLLL
EQC77430.1               LQTETTDLSLMLEQLTFEFLPLLEEKNLNWQLNLQKNVLATVDTEKIARVFDNLIRNAINYSYPDSPLLL
WP_103300092.1           LQTETTDLSLMLEQLTFEFLPLLEEKNLNWQLNLQKNVLATVDTEKIARVFDNLIRNAINYSYPDSSLLL
WP_113850318.1           LQTETTDLSLMLEQLTFEFLPLLEEKNLNWQLNLQKNVLATVDTEKIARVFDNLIRNAINYSYPDSPLLL
OQO78677.1               LQTETTDLSLMLEQLTFEFLPLLEEKNLNWQLNLQKNVLATVDTEKIARVFDNLIRNAINYSYPDSPLLL
WP_063856743.1           LQTETTDLSLMLEQLTFEFLPLLEEKNLNWQLNLQKNVLATVDTEKIARVFDNLIRNAINYSYPDSPLLL
WP_115241663.1           LQTETTDLSLMLEQLTFEFLPLLEEKNLNWQLNLQKNVLATVDTEKIARVFDNLIRNAINYSYPDSPLLL
WP_161667381.1           LQTETTDLSLMLEQLTFEFLPLLEEKNLNWQLNLQKNVLATVDTEKIARVFDNLIRNAINYSYPDSPLLL
                         **************************:*:***::**:* *******: *******:*******.: .: *

                         278         G1 box           F box           G2 box
VanS_C (this work)       ELVESDSIHIRLTNRGKTIPEEMIGRLFEPFYRMDSSRATATSGTGLGLPIAKEILLASGGDISAESKDE
WP_081116391.1           AISESDDILIRLTNQGKTIPAEKIGLIFEPFYRMDASRATATGGTGLGLPIAKEIVEALGGKIWAESRDE
EOH76242.1               AISESDDILIRLTNQGKTIPAEKIGLIFEPFYRMDASRATATGGTGLGLPIAKEIVEALGGKIWAESRDE
WP_016608740.1           AISESDDILIRLTNQGKTIPAEKIGLIFEPFYRMDASRATATGGTGLGLPIAKEIVEALGGKIWAESRDE
EEV40576.2               AISESDDILIRLTNQGKTIPAEKIGLIFEPFYRMDASRATATGGTGLGLPIAKEIVEALGGKIWAESRDE
WP_005228833.1           AISESDDILIRLTNQGKTIPAEKIVLIFEPFYRMDASRATATGGTGLGLPIAKEIVEALGGKIWAESRDE
WP_034869172.1           AISESDDILIRLTNQGKTIPAEKIVLIFEPFYRMDASRATATGGTGLGLPIAKEIVEALGGKIWAESRDE
WP_142968727.1           AISESDDILIRLTNQGKTIPAEKIGLIFEPFYRMDASRATATGGTGLGLPIAKEIVEALGGKIWAESSDE
GEB29600.1               AISESDDILIRLTNQGKTIPAEKIGLIFEPFYRMDASRATATGGTGLGLPIAKEIVEALGGKIWAESRDE
WP_156236642.1           AISESDDILIRLTNQGKTIPAEKIGLIFEPFYRMDASRATATGGTGLGLPIAKEIVEALGGKIWAESRDE
WP_142964727.1           AISESDDILIRLTNQGKTIPAEKIGLIFEPFYRMDASRATATGGTGLGLPIAKEIVEALGGKIWAESRDE
WP_142973537.1           AISESDDILIRLTNQGKTIPAEKIGLIFEPFYRMDVSRATATGGTGLGLPIAKEIVEALGGKIWAESRDE
WP_142437529.1           AISESDDILIHLTNQGKTISAEKIGLIFEPFYRMDASRATATGGTGLGLPIAKEIVEALGGKIWAESRDE
WP_142962782.1           AISASDDILIRLTNHGKTISAEKIALIFEPFYRMDASRATATGGTGLGLPIAKEIVEALGGKIWAESRDE
WP_081118442.1           AISASDDILIRLTNHGKTIPAEKIALIFEPFYRMDASRATATGGTGLGLPIAKEIVEALGGKIWAESRDE
WP_140965761.1           AISASDDILIRLTNHGKTIPAEKIGLIFEPFYRMDASRATATGGTGLGLPIAKEIVEALGGKIWAESRDE
WP_096742870.1           AISASDVILIRLTNHGKTIPAEKIGLIFEPFYRMDASRATATGGTGLGLPIAKEIVEALGGKIWAESRDE
OOG26042.1               AISASDDILIRLTNHGKTIPAEKIGLIFEPFYRMDASRATATGGTGLGLPIAKEIVEALGGKIWAESRDE
WP_161642824.1           SISESDDILIHLTNHGKTIPDEKLALIFEPFYRMDASRASATGGTGLGLPIAKEIVEALGGKIWAESCDE
WP_086269313.1           ELVESDSIHIRLTNRGKTIPEEMIGRLFEPFYRMDSSRATATGGTGLGLPIAKEILLASGGDISAESKDE
WP_155853362.1           ELVESDSIHIRLTNRGKTIPEEMIGRLFEPFYRMDSSRATATGGTGLGLPIAKEILLASGGDISAESKDE
EQC77430.1               ELVESDSIHIRLTNRGKTIPEEMIGRLFEPFYRMDSSRATATGGTGLGLPIAKEILLASGGDISAESKDE
WP_103300092.1           ELVESDSIHIRLTNRGKTIPEEMIGRLFEPFYRMDSSRATATGGTGLGLPIAKEILLASGGDISAESKDE
WP_113850318.1           ELVESDSIHIRLTNRGKTIPEEMIGRLFEPFYRMDSSRATATGGTGLGLPIAKEILLASGGDISAESKDE
OQO78677.1               ELVESDSIHIRLTNRGKTIPEEMIGRLFEPFYRMDSSRATATGGTGLGLPIAKEILLASGGDISAESKDE
WP_063856743.1           ELVESDSIHIRLTNRGKTIPEEMIGRLFEPFYRMDSSRATATGGTGLGLPIAKEILLASGGDISAESKDE
WP_115241663.1           ELVESDSILIRLTNRGKTIPEEMIGRIFEPFYRMDSSRATATGGTGLGLPIAKEILLASGGDISAESKDE
WP_161667381.1           ELVESDSILIRLTNRGKTIPEEMIGRIFEPFYRMDPSRATATGGTGLGLPIAKEILLASGGDISAESKDE
                         :   ** * *:***:****. * :  :******** ***:**.************: * **.* *** **

                         348
VanS_C (this work)       TIIFNVRLPKPANN-------------------
WP_081116391.1           TIVFSIRLPKAAMEKNEQLSKPHPSEPTA----
EOH76242.1               TIVFSIRLPKAAMEKNEQLSKPHPSEPTA----
WP_016608740.1           TIVFSIRLPKAAMEKNEQLSKPHPSEPTA----
EEV40576.2               TIVFSIRLPKAAMEKNEQLSKPHPSEPTA----
WP_005228833.1           TIVFSIRLPKAAMEKNEQLSKPHPSEPTA----
WP_034869172.1           TIVFSIRLPKAAMEKNEQLSKPHPSEPTA----
WP_142968727.1           TIVFSIRLPKAAMEKNEQLSKPHPSEPTA----
GEB29600.1               TIIFSIRLPKAAMEKNEQLSKPHPSEPTA----
WP_156236642.1           TIVFSICLPKAAMEKNEQLSNPHPSEPTA----
WP_142964727.1           TIVFSIRLPKAAMEKNEQLSKPYPSEPTA----
WP_142973537.1           TIVFSIRLPKAAMEKNEPLSKPHPSEPIA----
WP_142437529.1           TIVFSIRLPKVSMEKNEQRSKPHPSEPTA----
WP_142962782.1           TIVFSIRLPKAAMEKNEQRSKPHPSEPTS----
WP_081118442.1           TIVFSIRLPKAAMEKNEQRSKPHPSEPTS----
WP_140965761.1           TIVFSIRLPKAAMEKNEQRSKPHPSEPTS----
WP_096742870.1           TIVFSIRLPKAAMEKNEQRSKPHPSEPTS----
OOG26042.1               IIVFSIRLPKASLEKTKSLSELQTSEPTS----
WP_161642824.1           TIRFSIRLPKYSLGKVTLLSESNDSKDEKFPFF
WP_086269313.1           TIIFNVRLPKPANN-------------------
WP_155853362.1           TIIFNVRLPKPANN-------------------
EQC77430.1               TIIFNVRLPKPANN-------------------
WP_103300092.1           TIIFNVRLPKPANS-------------------
WP_113850318.1           TIIFNVRLPKPANS-------------------
OQO78677.1               TIIFNVRLPKPANS-------------------
WP_063856743.1           TIIFNVRLPKPANS-------------------
WP_115241663.1           TIIFNVRLPKPANS-------------------
WP_161667381.1           TIIFNVRLPKPANS-------------------
                          * *.: *** :
```

Figure S10. Sequence conservation within type-C CA-domain sequences. Shown is the Clustal Omega alignment (54) of 27 VanS$_C$ sequences (9), together with the sequence corresponding to the structure reported in this paper. The numbering shown is for the structure reported in this paper. The sequence-conservation key is the same as that used for Fig. S9.

VanA	IEFK**N**TGS-
VanB	LSVL**N**MGVH
VanC	IRLT**N**RGKT
VanD	IRLT**N**RGKT
VanE	VSVG**N**ITDK
VanG	LKFI**N**HSNM
VanL	FKIT**N**RTYT
VanM	VQVV**N**YGDS
VanN	LKIT**N**ETHT
S. coelicolor	LVVE**N**TGDL
A. teicomyceticus	LTVE**N**TGEQ
S. toyocaensis	LSVE**N**TGEV

Figure S11. Conservation of the Asp-to-Asn mutation in VanS sequences. Shown are G1-box sequences from VanS proteins associated with different VRE genotypes, as well as three VanS proteins from non-Enterococcal species. All contain an asparagine residue (highlighted in red) in the G1 box, occupying a position normally filled by an aspartic acid in most sensor histidine kinases.

A.

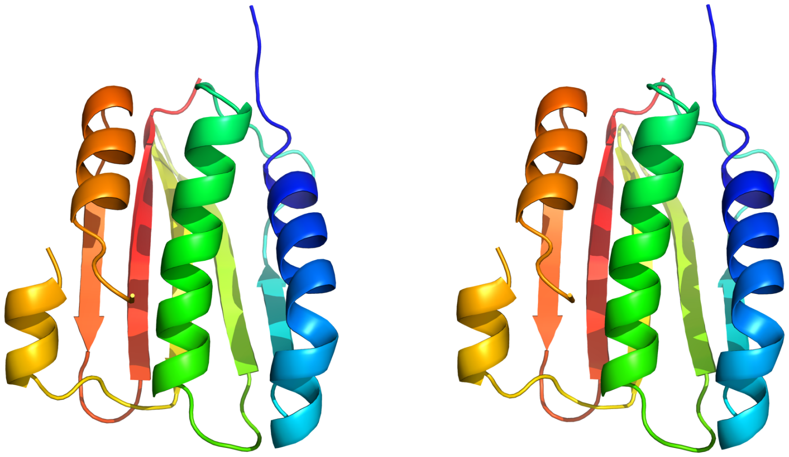

B.

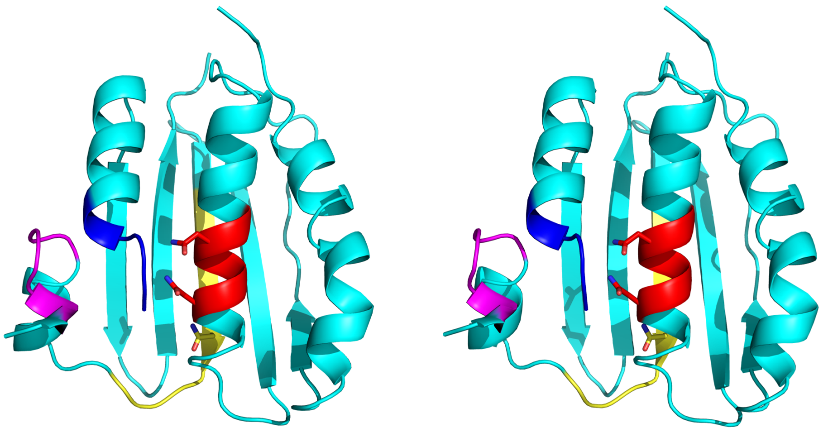

Figure S12: Stereo images of the VanS CA-domain proteins. Divergent stereo versions of panels B & C from Figure 1. (A) VanS$_A$ CA domain, shown in a rainbow color scheme (N-terminus = blue; C-terminus = red). (B) VanS$_C$ CA domain, with various functional regions highlighted. N-box, red; G1-box, yellow; F-box, magenta; G2-box, blue.

Table S1. Primers used for subcloning of CA-domain constructs.

Primer	Sequence (5' to 3')
$VanS_A$ CA domain forward	AGATTGGTGGCCAAACCATTACGCTGACCAAAAC
$VanS_A$ CA domain reverse	GAGGAGAGTTTAGACATTAGTCAACCAGATCCGGCATTGC
$VanS_C$ CA domain forward	AGATTGGTGGCGGTCTGCAGACGGAAACCACGGAT
$VanS_C$ CA domain reverse	GAGGAGAGTTTAGACATTAATTGTTGGCCGGTTTCGGCAG
EnvZ CA domain forward	CCGCGAACAGATTGGTGGCGGTCGCACCGGACAGGAGATGCC
EnvZ CA domain reverse	CTTCTCGAGGAGAGTTTAGACGATTACCCTTCTTTTGTCGTGCTCTG

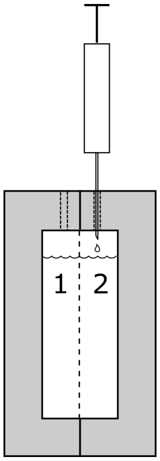

Derivation of Binding Expression for Equilibrium Dialysis Experiments

Given a cell with two chambers, separated by a dialysis membrane permeable to ligand but not to protein.

Let V_1 = volume of chamber #1, V_2 = volume of chamber #2 (for the apparatus used in this paper, the volumes of the two chambers are equal, thus $V_1 = V_2$).

Initiate the experiment by injecting protein (or buffer) into chamber #1 and ligand into chamber #2. After equilibrium is reached, read out free ligand concentration in chamber #2.

L_0 = initial concentration of ligand that is injected into chamber #2

P = concentration of protein in chamber #1

L_f = concentration of free ligand at equilibrium

P_f = concentration of free protein at equilibrium

PL = concentration of protein-ligand complex at equilibrium

At equilibrium:

$$L_0 \cdot V_2 = L_f \cdot V_1 + L_f \cdot V_2 + PL \cdot V_1$$ *(Eq. 1: Conservation of mass for ligand)*

Since $V_1 = V_2$, volumes cancel:

$$L_0 = 2L_f + PL$$

$$PL = L_0 - 2L_f$$ *(Eq. 1.1)*

$$P = P_f + PL$$ *(Eq. 2: Conservation of mass for protein)*

Rearrange Eq. 2 and substitute Eq. 1.1:

$$P_f = P - PL = P - \left(L_0 - 2L_f\right) = P - L_0 + 2L_f$$ *(Eq. 2.1)*

$$K_d = \frac{P_f \cdot L_f}{PL}$$ *(Eq. 3: Equilibrium expression)*

$$K_d = \frac{(P - L_0 + 2L_f)(L_f)}{L_0 - 2L_f}$$ *(Plug Eq. 2.1 into equilibrium expression)*

$$K_d \cdot L_0 - 2K_d \cdot L_f = P \cdot L_f - L_0 \cdot L_f + 2{L_f}^2$$

$$2{L_f}^2 - (L_0 - P - 2K_d)L_f - K_d \cdot L_0 = 0$$

$$L_f = \frac{(L_0 - P - 2K_d) + \sqrt{(L_0 - P - 2K_d)^2 + 8K_d \cdot L_0}}{4}$$ *(positive root is the correct one)*
